# MyBioScope: a new frontier in gut microbiome and health research

**DOI:** 10.1186/s40643-026-01044-1

**Published:** 2026-05-23

**Authors:** Kristina Žukauskaitė, Angela Horvath, Selina Tripolt, Hansjörg Habisch, Tobias Madl, Christian Pacher-Deutsch, Maximilian Nepel, Irina Balazs, Vanessa Stadlbauer

**Affiliations:** 1 Division for Gastroenterology and Hepatology, Department of Internal Medicine, Medical University of Graz, Graz, Austria; 2 Division Translational Precision Medicine, Center for Biomarker Research in Medicine (CBmed GmbH), Graz, Austria; 3 Division of Medical Psychology, Psychosomatics and Psychotherapeutic Medicine, Medical University of Graz, Graz, Austria; 4 Division Medicinal Chemistry, Otto Loewi Research Center, Medical University of Graz, Graz, Austria; 5 BioTechMed-Graz, Graz, Austria

**Keywords:** MyBioScope, Gut microbiome, In vitro model, Bioreactor system, Oralization, Sarcopenia, Modelling

## Abstract

Disruptions in the gut microbiome are linked to various diseases, but their roles in conditions such as age-related muscle loss (sarcopenia) and drug-induced microbial changes remain poorly understood. To address this gap, *MyBioScope*, a novel in vitro model using the DASbox^®^ mini bioreactor system and human stool samples, was developed to simulate the anaerobic environment of the gastrointestinal (GI) tract. Bioreactors containing 120–200 mL of cultivation media were inoculated with stool slurry, stabilized over 24 h, and maintained with a customizable feeding protocol for multi-day experiments. Samples were analyzed using *16S rRNA* gene sequencing, quantitative PCR, and metabolomics. Four pilot studies were conducted to validate the platform and model specific disease states, including proton pump inhibitor-induced GI tract oralization and microbiome alterations associated with sarcopenia. The workflow incorporated an anaerobic stool collection kit for user-friendly, room-temperature sample transport and storage. Our results demonstrated consistent microbial community structure and metabolic activity within disease-mimicking conditions. *MyBioScope* enabled reproducible, controlled studies of gut microbial dynamics and provided a scalable tool for investigating disease-specific microbiome changes. This platform may support translational efforts to integrate microbiome insights into clinical research, therapeutic development, and personalized medicine. In conclusion, this novel bioreactor-based in vitro model, MyBioScope, shows strong potential for in-depth exploration of disease-specific microbiomes and can facilitate new ways for integrating the knowledge of the microbiome’s impact on human health and disease into clinical practice.

**Graphical abstract:**

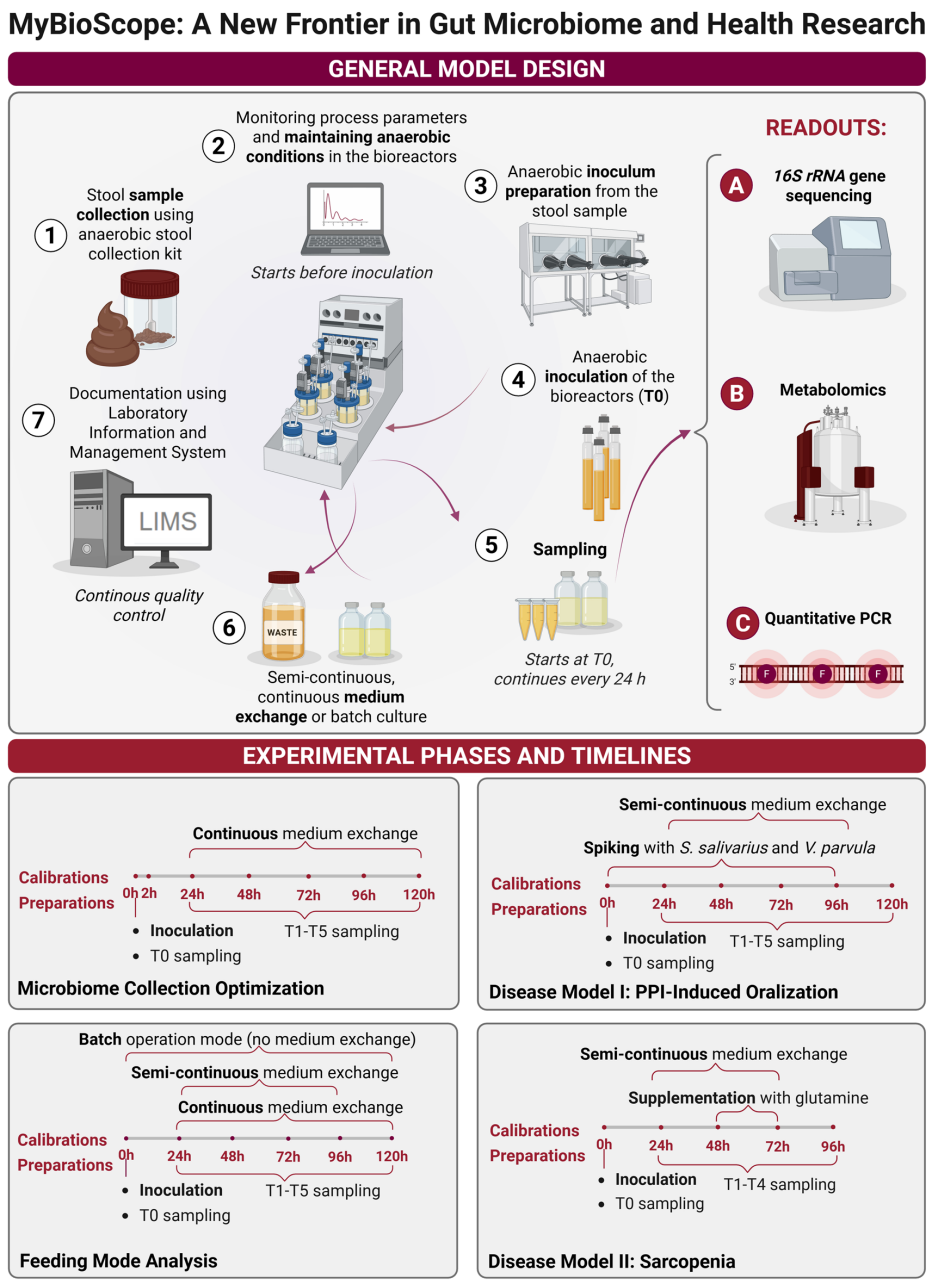

**Supplementary Information:**

The online version contains supplementary material available at 10.1186/s40643-026-01044-1.

## Introduction

The human microbiome, comprising 10^13^–10^14^microbial symbionts, is predominantly represented by gastrointestinal (GI) bacteria (Turnbaugh et al. 2007). Dysbiosis can contribute to disease onset and progression, making the gut microbiome a potential therapeutic target (Human Microbiome Project Consortium 2012; Zhao et al. 2023). While human clinical studies are the gold standard (Misra 2012), they are costly, time-consuming, and sometimes ethically or logistically constrained (Nguyen et al. 2015; Fogel 2018; Landi et al. 2021). Animal models, including transgenic mice, are commonly used, yet anatomical, genetic, and lifestyle differences limit translation (Nguyen et al. 2015).

In vitro systems offer controlled environments to study microbial interactions, but replicating the dynamic gut ecosystem using stool-derived inocula remains challenging (Žukauskaitė et al. 2024).

We introduce MyBioScope, a novel bioreactor-based in vitro model using stool samples and the DASbox^®^ mini bioreactor system. This model was optimized and validated through four studies: the first two focused on anaerobic sample collection and feeding mode effects on microbial composition, while the latter two assessed disease modelling and experimental interventions.

MyBioScope was adapted to model microbiomes associated with sarcopenia, the progressive loss of muscle mass and strength linked to frailty in aging populations (Cruz-Jentoft and Sayer 2019). Recent evidence indicates that sarcopenia correlates with reduced microbial diversity, loss of butyrate-producing taxa (e.g., Ruminococcaceae, Lachnospiraceae), and enrichment of pro-inflammatory genera (Song et al. 2024; Zhang et al. 2024b; Lapauw et al. 2024). Using the model, we assessed the effects of twice-daily L-glutamine supplementation, an amino acid critical for protein synthesis and muscle growth (Newsholme et al. 2003; Cruzat et al. 2018), on sarcopenia-specific and healthy microbiomes.

The model was further adapted to simulate oralization, the introduction of oral bacteria into the gut microbiome, which can result from long-term proton pump inhibitor (PPI) use or surgery (Horvath et al. 2019, 2021). Oralization is characterized by *Streptococcus* enrichment, loss of gut commensals, and taxonomic shifts, which may compromise the intestinal barrier, trigger immune responses, and promote dysbiosis (Kiecka and Szczepanik 2023; Fossmark and Olaisen 2024; Xiao et al. 2024; Zhang et al. 2024a). In vitro modelling of oralization offers insights into microbial shifts under prolonged perturbations and may guide therapeutic strategies to restore gut health.

## Methods

### Study design and ethics

This research was conducted at the Medical University of Graz, Graz, Austria following the approval of the local Ethics Committee (protocol No. 34-323 ex 21/22 1126/2022). All participants were ≥ 18 years and provided written informed consent. The MyBioScope model was developed in two phases: (1) establishment and optimization of the in vitro gut microbiome system, and (2) application to disease modelling with or without experimental interventions.

### Study participants

Six healthy volunteers without GI complaints were included to establish the in vitro model. Three females (25–30 years) participated in anaerobic sample collection and the PPI-induced oralization scenario, and three additional participants (two females, one male, 26–31 years) in feeding mode experiments. Exclusion criteria included recent antibiotic, prebiotic, or probiotic use, and diarrhea. For the sarcopenia scenario, three patients (two males, one female, 25–70 years) diagnosed per European Working Group on Sarcopenia in Older People criteria (Cruz-Jentoft et al. 2010) were included (25–70 years), along with three healthy controls (two males and one female, aged 28–32).

### Technical setup of the MyBioScope model

**Fig. 1:**
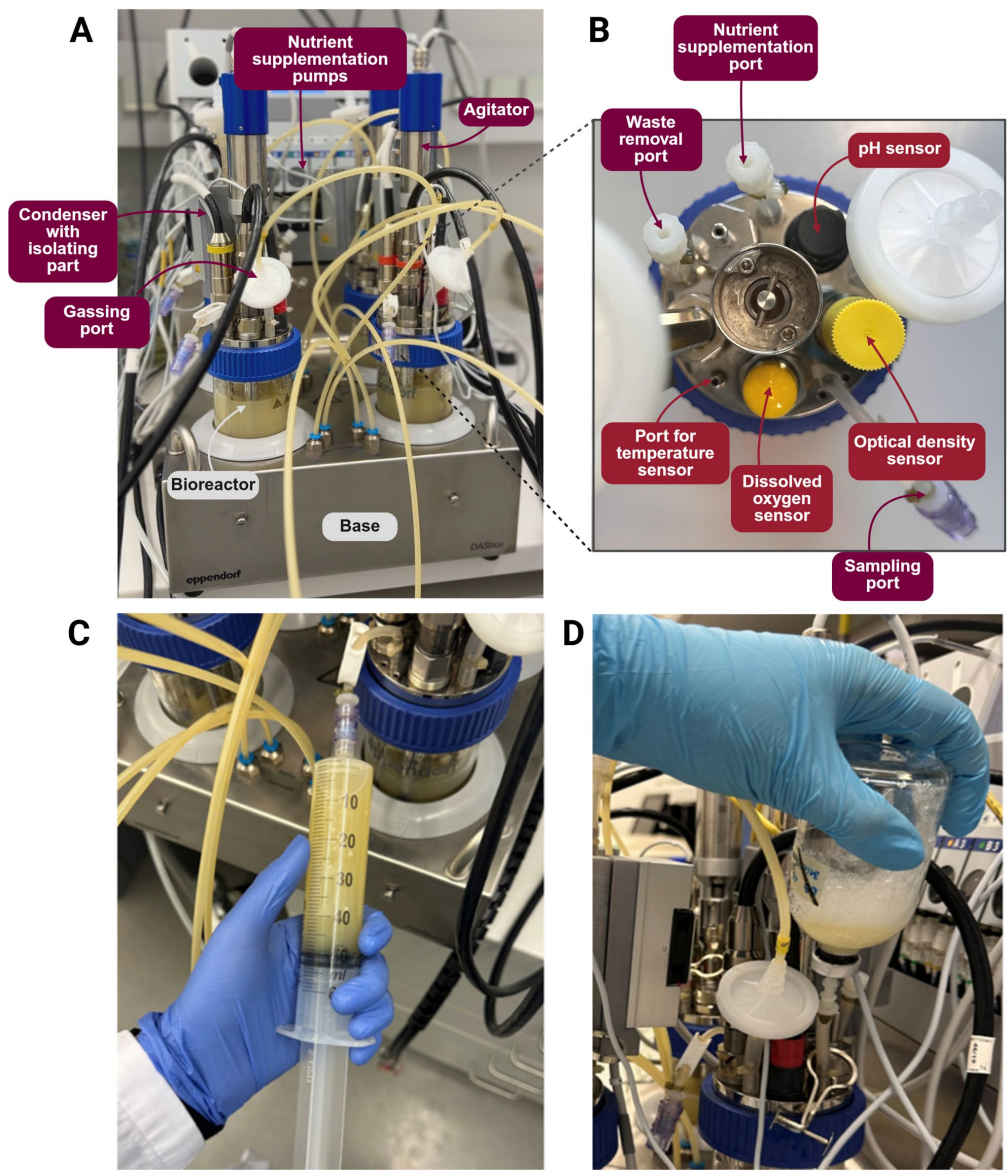
Technical setup of the in vitro human gut microbiome model MyBioScope based on the DASbox^®^ mini bioreactor system and the process of anaerobic semi-continuous medium exchange. (A) A frontal view of the base shows the bioreactor vessels connected to the base, sensors, and gassing tubing. (B) Top view of a bioreactor vessel, illustrating with the sensors used in this setup. (C) Removal of the bacterial culture from the bioreactors and (D) anaerobic supplementation with fresh medium through a needle that is attached to the nutrient supplementation port. The flow of medium from the glass flask into the bioreactors was accomplished due to pressure differences between the flask and the bioreactors. Consequently, there is no oxygenation throughout this process. Original pictures were modified using BioRender.com

The MyBioScope model used the DASbox^®^ mini bioreactor system (Eppendorf, Germany) with four glass vessels equipped with optical and electrochemical sensors for monitoring temperature, optical density (600 nm), pH, and dissolved oxygen (Fig. 1). Bryant and Burkey anaerobic culture medium (NutriSelect^®^ Plus, Merck, Germany, or custom medium (Table S1) supported a broad range of gut bacteria. Bioreactors were sterilized at 121 °C for 20 min, cooled, and purged with nitrogen to establish anaerobic conditions. Agitation was maintained at 200 rpm, with a working volume of 120–200 mL and starting pH 5.9 ± 0.2 (25 °C), and temperature held at 37 ºC. Dissolved oxygen was kept at 0% via submersed 100% nitrogen at 1 sL/h. pH was not actively controlled to preserve natural microbial interactions, and all parameters were monitored in real time using DASware^®^ control 5 software (Eppendorf, Germany). Technical specifications, materials, and reagents are detailed in Tables S2–S4.

### Optimization of stool collection and anaerobic inoculum preparation

Fresh stool samples were collected from participants using GutAlive^®^ anaerobic containers (MicroViable Therapeutics, Spain) and stored for < 1, 2, 24, or 48 h at room temperature. Samples were processed in a Whitley A85 anaerobic workstation (Don Whitley Scientific, UK) by mixing with Bryant and Burkey anaerobic culture medium, with a visible colour change confirming anaerobic conditions (Fig. S1).

Suspensions were centrifuged (180 × *g*, 10 min, 4 °C) to sediment insoluble material, and the supernatant was processed anaerobically by filtration through a 100 µm strainer (Corning^®^ 100 µm Nylon Cell Strainer, Corning, USA) and transferred to sterile glass tubes. Tubes were briefly gassed with nitrogen (15–60 s) using syringe filters (Nalgene^®^ Syringe Filter with SFCA Cellulose Acetate Membrane or Nalgene^®^ Syringe Filter, Sterile SFCA membrane, both Thermo Scientific, China) to create anaerobic pressure and inoculated into bioreactors via the nutrient port using a sterile needle (VACUETTE^®^ Luer Adapter 20G, Nipro).

Bioreactors were run for 120 h under continuous feeding with a retention time of 28.3 h (Haindl et al. 2021). Samples were collected every 24 h, and bacterial pellets were obtained by centrifugation (5000 rpm, 10 min, 4 °C). Pellets and supernatants were stored at –80 °C for downstream analyses. *16S rRNA* gene sequencing was used to assess the effects of storage time on microbial composition, diversity, and stability.

### Feeding mode analysis: supplementation of fresh nutrients

Four different nutrient (Bryant and Burkey’s anaerobic culture medium) supplementation strategies (*further*—feeding modes) were tested: (1) Batch feeding mode, where no culture medium was added or removed throughout cultivation time; (2) Continuous feeding mode with fresh nutrient flow (along with waste removal) at a rate of ~ 3 mL/h starting 24 h after inoculation; (3) Semi-continuous feeding mode with 50% medium exchange daily starting 24 h after inoculation; (4) Semi-continuous feeding mode with 25% medium exchange twice a day starting 24 h after inoculation, with an 8 h interval between medium exchanges. The semi-continuous feeding mode is illustrated in Fig. 1C, D. *16S rRNA* gene sequencing was conducted to assess the effect of different feeding modes on the microbiome composition, diversity, and stability over time.

### Establishment of the PPI-induced oralization model

The PPI-induced oralization model was established by regularly introducing oral bacteria—*Veillonella parvula* and *Streptococcus salivarius*—into the bioreactors, mimicking continuous oral bacterial swallowing in humans (Horvath et al. 2019). Oral bacteria were pre-cultivated overnight (*S. salivarius*) or for 48 h (*V. parvula*) due to differing growth rates (media composition in Table S5). Bacterial concentrations were determined using the QUANTOM™ Total Cell Staining Kit and QUANTOM Tx™ Microbial Cell Counter (Logos Biosystems, South Korea).

Spiking was integrated into a semi-continuous medium exchange once daily, with *V. parvula* and *S. salivarius* added at 0.2% and 0.6% of total bioreactor bacterial concentration, respectively. Four spiking strategies were chosen: (1) spiking once after inoculation; (2) spiking once 48 h after inoculation; (3) spiking every day, starting after inoculation; (4) spiking every day, starting 48 h after inoculation. Bioreactor samples were collected every 24 h over 120 h. DNA was extracted from bacterial pellets, and quantitative polymerase chain reaction (qPCR) was employed to ascertain the abundance of oral bacteria in the system.

### DNA extraction and qPCR

For the PPI-induced oralization model establishment, DNA was extracted from bacterial pellets by mixing them with 180 µL TRIS–HCl lysis buffer (composition in Table S6) and transferring them into 1.5 mL tubes (SC Micro Tube PCR-PT, SARSTEDT, Germany) containing glass beads (Assistent, Germany). Samples were homogenized using a MagNA Lyser (Roche, Penzberg, Germany) at 6500 × g for 45 s. DNA was then isolated using the DNeasy Blood & Tissue Kit (QIAGEN, Valencia, CA, USA) following the manufacturer’s protocol. DNA purity and concentration were determined with a NanoDrop™ 2000/2000c spectrophotometer (Fisher Scientific, USA), and working concentrations were adjusted to 2.5 ng/µL.

The qPCR was performed using the Bio-Rad CFX instrument and Bio-Rad CFX Maestro software (v1.1) from Bio-Rad, USA. Primer sequences used for oral bacteria detection, data normalization, composition of the PCR reaction mixtures, and temperature regimes are provided in Tables S7–9. All samples were run in duplicate to ensure reproducibility. Starting quantities (SQ) were calculated from standard curves generated from serial dilutions of pooled undiluted DNA samples and reported as relative, unitless values.

### Establishment of the sarcopenia model and glutamine supplementation

Gut microbiomes from healthy donors and patients with sarcopenia were cultivated for up to 96 h in Bryant and Burkey anaerobic medium under semi-continuous feeding conditions. Medium exchange began 24 h post-inoculation, and Kabi^®^ Glutamine Powder (Fresenius Kabi Deutschland GmbH, Germany) supplementation started at 48 h, with a daily dose of 1.25 g.

Considering an estimated colonic volume of ~ 400 mL (Sender et al. 2016) and that ~ 75% of orally ingested glutamine is absorbed in the upper GI tract (Déchelotte et al. 1991; Coster et al. 2004), approximately 25% reaches the colon. With a recommended oral intake of 20–30 g/day and a scaled 200 mL model, this corresponds to an equivalent daily intake of 10 g, representing ~ 12.5% colonic availability. Accordingly, the system received 1.25 g glutamine per day, administered in two 0.625 g doses at 8 h intervals.

Samples were collected every 24 h for DNA extraction and *16S rRNA* gene sequencing. Supernatants were frozen at − 80 °C and later analyzed by Nuclear Magnetic Resonance (NMR) spectroscopy to assess metabolomic changes. Detailed descriptions of NMR procedures are provided in the Supplementary Methods. Summary of all technical details of the study is provided in Table S10.

### Raw read processing and data normalisation

Amplicon sequencing of bacterial DNA was performed by the external facilities (see Supplementary Methods for detailed protocols). Raw read processing and downstream analysis were conducted in-house using QIIME 2 (Bolyen et al. 2019) on a local Galaxy instance. Read quality was assessed with FastQC and MultiQC, and forward and reverse reads were truncated according to the quality reports. Denoising was performed with DADA2 (Callahan et al. 2016), implemented in QIIME2. Taxonomic classification was assigned using a naïve Bayes classifier trained on the SILVA 132 database (99% OTU level).

For alpha and beta diversity analyses, normalized data sets were used, each rarefied to an appropriate sequencing depth depending on the part of the study. Details on sequencing depth, rarefaction, and truncation values are provided in Table S11.

### Statistical analysis

Processed sequencing data were analyzed in R (R Core Team, 2023, version 4.3.0) via RStudio using the *CBmed Microbiome Analysis Platform*. Data were analyzed using alpha and beta diversity metrics, Linear Discriminant Analysis Effect Size (LEfSe), and linear models. Statistical significance was assessed via Permutational Multivariate Analysis of Variance (PERMANOVA), with results considered significant at *p* < 0.05. A detailed description of the statistical methods is provided in Supplementary Methods.

## Results

### Anaerobic microbiome collection optimization

With this pilot study we aimed to optimize stool sampling conditions to preserve native microbiome composition using an anaerobic microbiome collection kit. Bioreactor process parameters were unaffected by stool storage times of < 1 h, 2 h, 24 h, or 48 h at room temperature (Fig. S2).

**Fig. 2:**
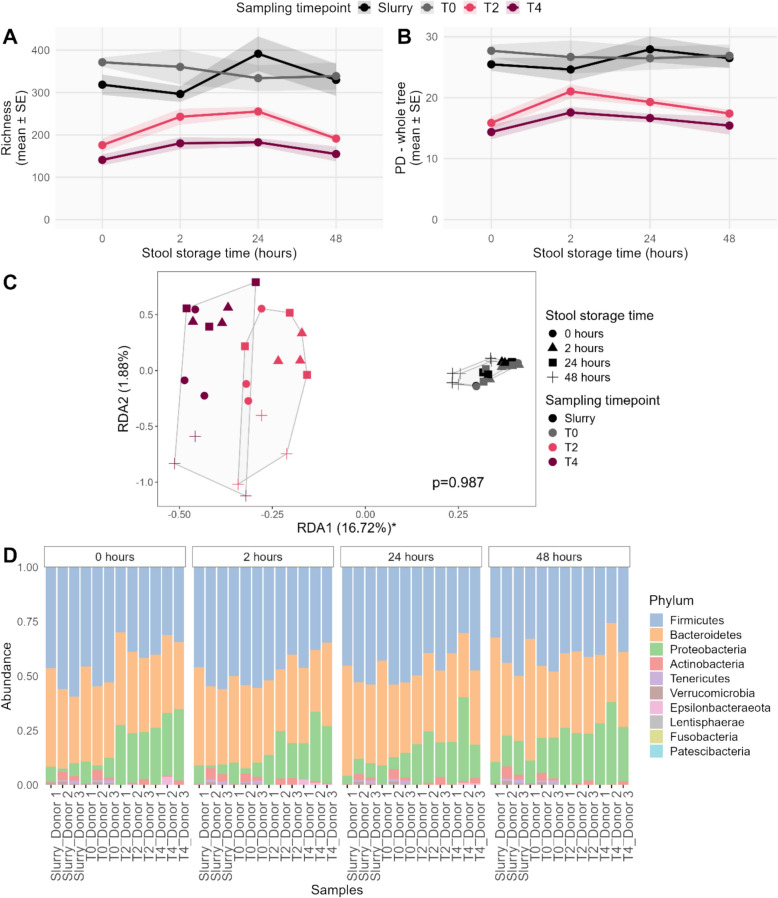
Changes in various alpha diversity indices across storage time (0, 2, 24, and 48 h) and cultivation time (Slurry – inoculum, T0 – sampling immediately after inoculation, T2 – sampling at 48 h after inoculation, T4 – sampling at 96 h after inoculation): **A** Species Richness, **B** Phylogenetic diversity (PD) of the samples stored in the microbiome collection kit containers for different time periods and cultured in the MyBioScope model for 96 h. Lines represent mean values; shaded areas indicate standard error (SE). **C** Redundancy analysis (RDA) of the microbiome composition of the samples stored in the microbiome collection kit containers for different times (0–48 h, depicted in various shapes) and cultured in the MyBioScope model for 96 h. Symbols denote different storage time in the microbiome collection containers (circle, 0 h; triangle, 2 h; square, 24 h; plus sign, 48 h), while colours represent different sampling time points during cultivation (black, Slurry; grey, T0; pink, T2; dark red, T4). Convex hulls show grouping according to the sampling timepoint. RDA1 and RDA2 represent the first and second axes of redundancy analysis, summarizing the relationships between stool storage time, cultivation time, and microbial composition. Asterisk denotes statistically significant axis. **D** Bacterial composition of the gut microbiome shown as a relative abundance at the phylum level (top 10) in the groups of different stool storage times in anaerobic microbiome collection containers. Every bar represents the community composition of one sample

Alpha diversity indices—including species Richness, Shannon, Inverse Simpson, Evenness, and Phylogenetic Diversity (PD)—declined significantly over cultivation time (all *p* ≤ 0.002 but were independent of stool storage duration (all *p* ≥ 0.279; Fig. 2A, B, Table S12). Beta diversity analysis via Principal Coordinates Analysis (PCoA) showed significant microbial community shifts over cultivation time (all *p* = 0.001), with no effect from storage time inside collection containers (all *p* = 1.000; Table S13), which was further confirmed by Redundancy Analysis (RDA) (Fig. 2C).

At the phylum level, bacterial composition remained relatively stable across cultivation and storage times, with similar profiles observed from all collection containers throughout the experiment (Fig. 2D).

### Impact of feeding modes on the gut microbiome

We assessed how different nutrient supplementation strategies influenced microbiome stability in the MyBioScope model. Bioreactor process parameters remained stable across feeding modes (Fig. S3). Semi-continuous feeding exhibited characteristic pH decreases during medium exchanges followed by recovery, while continuous feeding maintained the most stable pH.

**Fig. 3:**
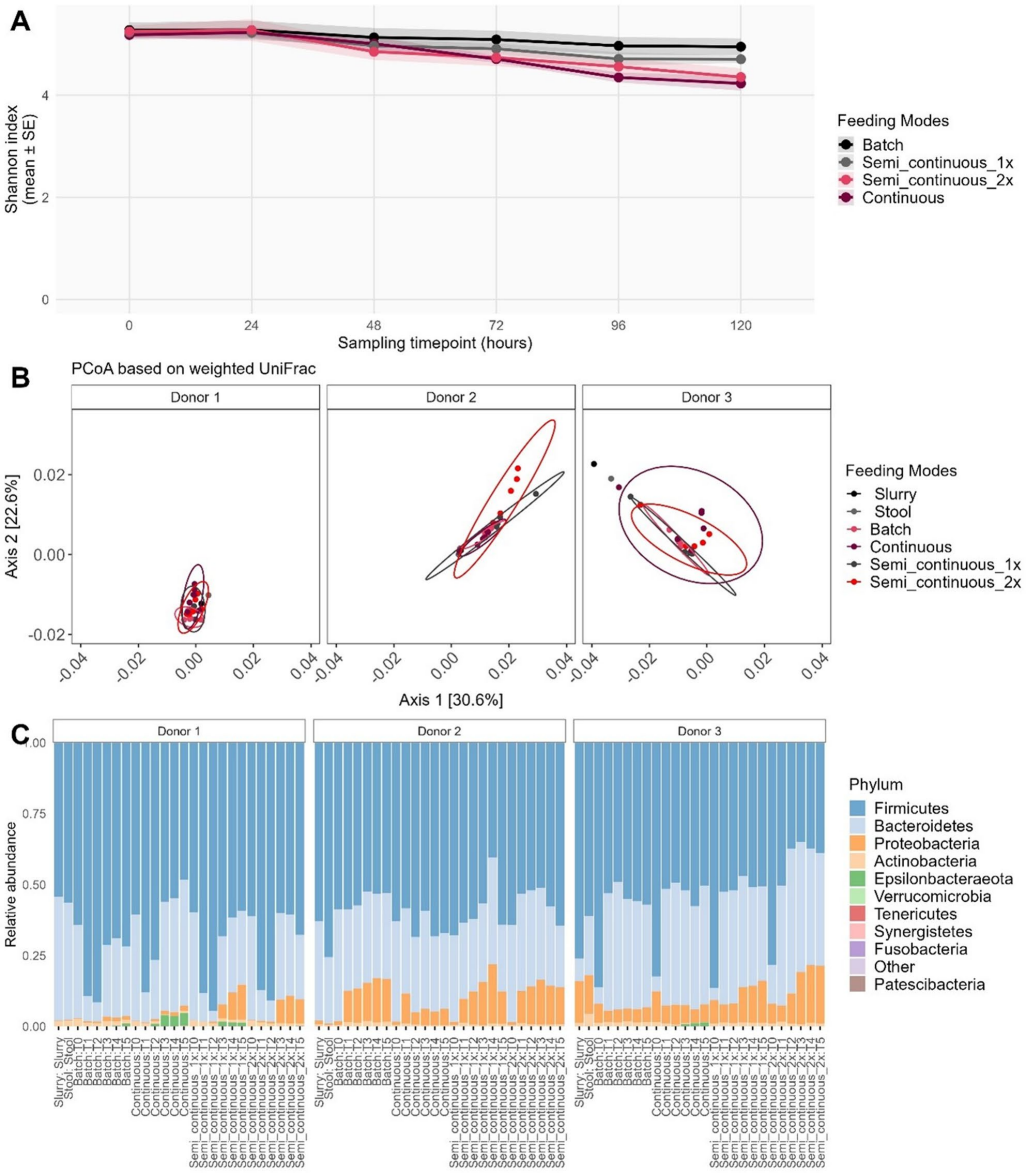
**A** Changes in Shannon index based on the operational feeding modes throughout the cultivation time in the MyBioScope model. Colours represent different feeding modes: batch, continuous, and semi-continuous (once or twice a day). Lines represent mean values; shaded areas indicate standard error (SE). **B** Microbiome beta diversity analyses of stool samples from healthy donors cultivated in the MyBioScope model for up to 120 h using different operational feeding modes (pink, Batch; dark red, Continuous; dark grey, Semi-continuous once a day; bright red, Semi-continuous twice a day, with an 8 h interval between nutrient supplementation). Distances based on weighted unique fraction metric were plotted via PCoA. **C** Bacterial composition of the gut microbiome shown as relative abundance at the phylum level (top 10) in the groups of different feeding modes in the MyBioScope model. Stool and slurry (inoculum) samples are presented as references. Every bar represents the community composition of one sample

Alpha diversity analysis indicated that batch feeding maintained the most stable microbiome composition over time. Among medium-exchange modes, semi-continuous once-daily exchange preserved stability better than semi-continuous twice-daily or continuous feeding. Across all samples, cultivation time significantly decreased Richness, Shannon, Inverse Simpson, and phylogenetic diversity indices (all *p* ≤ 0.030; Table S14. Within feeding modes, throughout the cultivation time continuous feeding led to significant decreases in Shannon (*p* < 0.001), evenness (*p* = 0.020), and phylogenetic diversity (*p* = 0.047), while semi-continuous twice-daily feeding also reduced Shannon (*p* = 0.001) and evenness (*p* = 0.030) indices over time (Fig. 3A).

Beta diversity analysis demonstrated that both feeding mode and experimental time significantly influenced microbiome composition (all *p* < 0.020, Table S15. Donor-dependent differences were observed, with each donor’s microbiome maintaining distinct compositions throughout cultivation, highlighting the reproducibility of the MyBioScope system (Fig. 3B).

*16S rRNA* gene sequencing revealed that microbiome composition at the phylum level remained relatively stable across feeding modes, with Firmicutes, Bacteroidetes, and Proteobacteria predominating, reflecting the compositions of stool, inoculum, and cultivated microbiomes (Fig. 3C).

### Disease scenario I: in vitro modulation of PPI-induced oralization

We adapted the MyBioScope model to simulate PPI-induced oralization by testing four bacterial spiking strategies aimed at steadily increasing *V. parvula* and *S. salivarius*: (1) a single spike immediately after inoculation; (2) a single spike 48 h post-inoculation; (3) daily spiking starting immediately after inoculation; and (4) daily spiking starting 48 h post-inoculation.

**Fig. 4:**
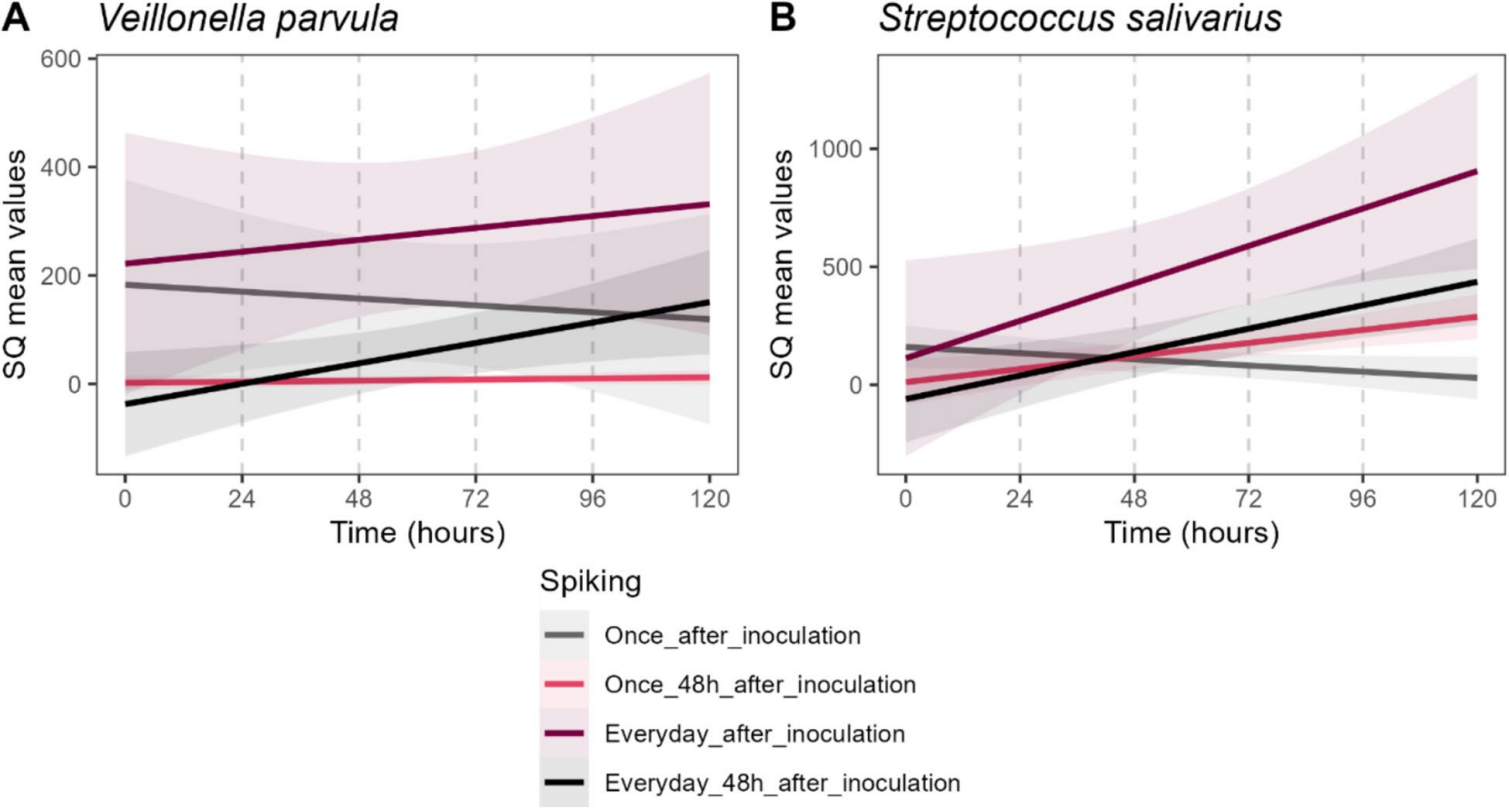
Changes in **A** *Veillonella parvula* and **B** *Streptococcus salivarius* abundance in the MyBioScope model based on spiking strategies. Results are presented as mean starting quantity (SQ) values. SQ values represent the relative starting quantity of target, calculated from the standard curve (unitless). Colours represent tested spiking strategies (and standard deviation), and grey dashed lines indicate semi-continuous medium exchange. Lines represent mean SQ values; shaded areas indicate standard error

Bioreactor process parameters demonstrated stable and consistent patterns throughout cultivation (Fig. S4). It was noted that the strategy of spiking only once, tended to steadily decrease the abundance of oral bacteria or keep it on a rather low concentration. Spiking bacteria every day, initiated immediately after inoculation, simulated a regular introduction of oral bacteria and yielded a steady increase and the highest general abundances throughout the entire experimental time (Fig. 4, Table S16).

### Disease scenario II: in vitro modulation of sarcopenia-specific gut microbiome

We applied the MyBioScope model to simulate sarcopenia-specific gut microbiome. Stool samples from patients with clinically diagnosed sarcopenia and healthy volunteers were cultivated for 96 h, with each sample inoculating four bioreactors: two supplemented with glutamine 48 h post-inoculation, and two serving as controls. Bioreactor process parameters remained stable throughout the experiment. Glutamine supplementation decreased pH and increased optical density in both healthy and sarcopenia groups (Fig. S5).

**Fig. 5:**
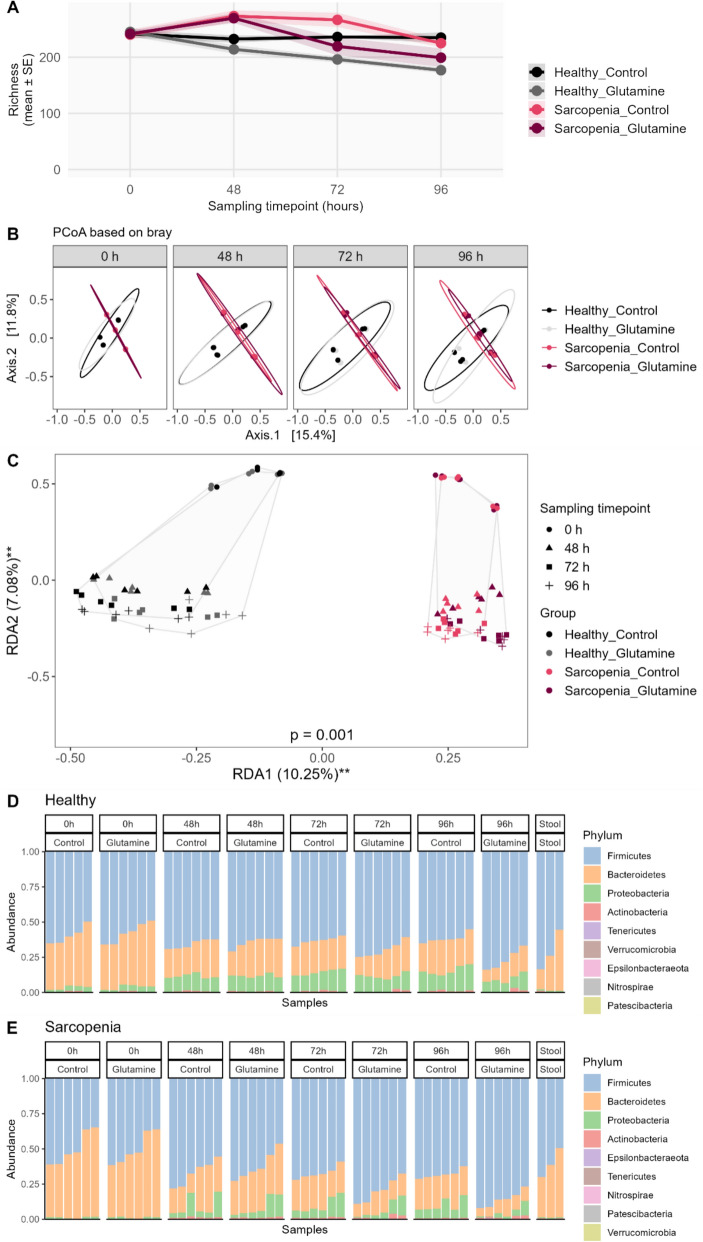
**A** Changes in the microbiome species richness of stool samples from sarcopenia patients and healthy donors with or without glutamine supplementation. Samples were cultivated in the MyBioScope model for 96 h. Lines represent mean values; shaded areas indicate standard error (SE). **B** Changes in the microbiome beta diversity of the stool samples from sarcopenia patients and healthy donors cultivated with or without glutamine supplementation in the MyBioScope model for up to 96 h. Distances based on unique fraction metric, weighted unique fraction metric, Bray–Curtis, and Jaccard dissimilarity were plotted via PCoA. **C** RDA plot showing microbiome beta diversity differences between sampling timepoints and bioreactor samples from healthy and sarcopenic individuals cultivated with or without glutamine supplementation along the significant RDA axes. Symbols denote different sampling timepoints (circle, 0 h; triangle, 48 h; square, 72 h; plus sign, 96 h), while colours represent different study groups (black, Healthy controls; grey, Healthy controls with glutamine supplementation; pink, Sarcopenia patients; dark red, Sarcopenia patients with glutamine supplementation). Convex hulls show grouping according to the study group. Asterisks denote statistically significant axes. **D–E** Gut microbiome composition at the phylum level (top 10) in samples from healthy donors and sarcopenia patients using the MyBioScope model. Stool samples are included as reference controls in the final panel. Each bar represents the microbial community of one sample, with all samples analyzed in duplicate

Alpha diversity analysis revealed significant differences between glutamine-treated and control microbiomes over time (Fig. 5A, Table S12). Richness decreased with glutamine supplementation in both groups (*p* < 0.002), with a more pronounced decline in healthy microbiomes. Glutamine also reduced the Shannon index in healthy (*p* = 0.041) and sarcopenia (*p* = 0.011) microbiomes, while Inverse Simpson index was unaffected. Evenness decreased significantly in glutamine-treated sarcopenia microbiomes (*p* = 0.026), and phylogenetic diversity declined in both healthy and sarcopenia groups (*p* < 0.0001 and *p* = 0.012, respectively).

Beta diversity analysis demonstrated significant differences between healthy and sarcopenia microbiomes (all *p* = 0.001), which persisted throughout cultivation. Glutamine supplementation further altered microbiome composition over time (Fig. 5B, Table S17).

RDA confirmed clear separation between healthy and sarcopenic microbiomes along the first RDA axis (*p* = 0.001), independent of cultivation time (Fig. 5C). Permutation testing showed that cultivation time also significantly influenced microbiome composition (*p* = 0.001), with samples at 0 h clustering separately from those collected at 48–96 h. Phylum-level microbiome compositions for healthy and sarcopenia groups are shown in Fig. 5D–E.

Heatmap analysis (Fig. S6) qualitatively indicated a distinct metabolomic shift following glutamine supplementation of sarcopenia-specific microbiomes, characterized by increases in fructose, formic acid, lactic acid, glutamine, succinic acid, galactose, valeric acid, capric acid, trimethylamine, and butyric acid, alongside decreases in metabolites such as acetic acid and fumaric acid.

## Discussion

In this study, we present MyBioScope, a novel in vitro gut microbiome model developed using the DASbox^®^ mini bioreactor system. The primary objectives were to optimize stool collection under anaerobic conditions, ensure microbiome stability during cultivation, and demonstrate the model’s adaptability for simulating disease-associated microbiomes, including PPI-induced oralization and sarcopenia.

The optimized protocol confirmed that GutAlive^®^ anaerobic collection containers effectively preserved native microbial composition for up to 48 h at room temperature, enabling flexible and user-friendly sampling suitable for clinical and translational studies. Subsequent optimization of feeding strategies revealed that batch feeding maintained the highest alpha diversity, while a semi-continuous once-daily feeding mode provided comparable stability with the additional advantage of daily supplement administration. This configuration supports long-term cultivation and dynamic interventions, making it particularly suited for clinical research applications.

The model was next adapted to simulate PPI-induced oralization, using *V. parvula* and *S. salivarius* as biomarkers (Horvath et al. 2019). To understand this process is critical, as suppression of gastric acid using PPIs allows oral bacteria to survive transit through the stomach and colonize the intestine, directly disrupting the intestinal barrier integrity. This ectopic colonization increases intestinal permeability and triggers inflammation (Zhang et al. 2026). Our model showed that among several spiking strategies tested, the daily introduction of oral bacteria from the onset of cultivation yielded the most stable colonization dynamics, closely reflecting the continuous physiological translocation of oral taxa via saliva. Compared with other oralization models (Etienne-Mesmin et al. 2023), MyBioScope offers a standardized and streamlined approach that avoids the need for saliva-based inoculation, thereby reducing experimental variability and improving reproducibility. While the absence of a mucus compartment remains a limitation in our model, this feature can be integrated in future developments.

Next, MyBioScope was applied to model sarcopenia-associated microbiomes. Stool samples from patients with sarcopenia and healthy donors maintained distinct community structures throughout cultivation, consistent with previous reports showing persistent microbiome and metabolome differences between these groups (Zhou et al. 2023). Glutamine supplementation further modulated the sarcopenia-associated microbiome, resulting in reproducible metabolomic shifts indicative of altered amino acid turnover and short-chain fatty acid production, suggesting that glutamine availability directly influences microbial metabolic pathways in sarcopenic conditions. Lactate, previously regarded solely as a byproduct of anaerobic glycolytic activity, is now acknowledged as a viable energy source in skeletal muscles (Lund et al. 2018). The observed elevation of this metabolite suggests the potential for a supplementary energy reservoir within the muscles provided by the microbiome. Although, an increase in glutamine concentrations was expected due to L-glutamine supplementation an increase was exclusively observed in the group affected by sarcopenia, which is suggesting a less efficient metabolism of L-glutamine by the dysbiotic microbiome. Notably, succinic acid plays a vital role in regulating muscle function. Upon secretion, succinate signals through its cognate receptor SUCNR1 in non-myofibrillar cells within muscle tissue to govern transcriptional programs responsible for muscle remodelling. The succinate-SUCNR1 signalling is crucial for the paracrine regulation of muscle innervation, muscle matrix remodelling, and muscle strength in response to exercise training. These findings suggest that L-glutamine supplementation may augment the beneficial muscle remodelling induced by succinate supplementation (Reddy et al. 2020). Also, it is known that succinate plays important role for intestinal epithelial barrier function and resistance to colonization by invading pathogens (Connors et al. 2019; Li et al. 2019). Interestingly, there was an increase in fructose and galactose after the supplementation with L-glutamine in the microbiome group from sarcopenia patients. Glutamine metabolism possibly indirectly influences other carbohydrates metabolism and even though it is the secondary effect of glutamine supplementation, cell culture studies showed that the galactose improved myotube’s glucose metabolism and metabolic switching, which could improve the growth of the muscles (Kase et al. 2013). Interestingly, there was an increase of butyric acid, which not only acts as a primary energy source for colonocytes, strengthening the gut barrier, but it also directly influences muscle mass (Davis et al. 2021). Animal study showed that by butyrate acts as a histone deacetylase inhibitor, which increases the expression of genes involved in mitochondrial biogenesis and enhances the anabolic signalling pathways necessary for maintaining muscle mass (Walsh et al. 2015). Similar effects of glutamine on microbial metabolites have been observed in other animal models (Yan et al. 2020; Li et al. 2023), and declining glutamine metabolism has been linked to age-related gut dysfunction (Meynial-Denis 2016). Together, these results highlight MyBioScope’s potential for studying disease-specific microbiome modulation and nutrient–microbe interactions under controlled in vitro conditions.

Various bioreactor-based systems are currently available for modulating and studying the gut microbiome; however, MyBioScope distinguishes itself through its rapid stabilization, shortened experimental duration, and high degree of adaptability regarding inoculum selection and supplementation.

A primary advantage of the MyBioScope system is its 24-h stabilization period. Following this brief window, various interventions—such as medium exchange or specific supplementation—can be implemented, as demonstrated in the current study. This rapid equilibration significantly reduces the total experimental timeline, typically requiring only five days, though this remains flexible based on the specific research question. In contrast, more established models often require much more extensive timeline. For instance, the Simulator of Human Intestinal Microbial Ecosystem (SHIME^®^) typically necessitates a stabilization period of 10–20 days and a total experimental duration of 8–10 weeks (Van de Wiele et al. 2015). While the mucosal adaptation of this system (M-SHIME^®^) offers an experimental window more comparable to MyBioScope (Van de Wiele et al. 2015), its application is specifically tailored toward microbial colonization of mucosal surfaces, which limits its broader utility in general luminal studies (Van den Abbeele et al. 2013). Similarly, the Polyfermentor Intestinal Model (PolyFermS) requires approximately six days for stabilization and a total experimental run of at least 38 days (Zihler Berner et al. 2013). While the TNO in vitro model of the colon (TIM-2) achieves a short stabilization period similar to MyBioScope, evidence suggests that the microbiome in the TIM-2 model undergoes significant compositional shifts during this phase as it adapts to the specific feed and vessel environment (Venema 2015).

While the MyBioScope system is compatible with various inoculum sources, the preferred methodology involves utilizing stool samples from individual donors to better represent natural diversity. By inoculating the system with samples from different individuals, it is possible to capture critical biological variability and enhance the reliability of the observations, mirroring the validated approaches used in the SHIME^®^ and TIM-2 systems (Van de Wiele et al. 2015; Venema 2015). In a typical experimental design, multiple donors are recruited, and the protocol is repeated with each unique sample to ensure the biological relevance and reproducibility of the findings. Despite this preference for individual variability, the system maintains the inherent flexibility to utilize pooled stool samples when necessary. This allows researchers to strategically tailor the inoculation protocol to meet specific experimental objectives, whether the goal is to observe individualized responses or to establish a standardized microbial baseline.

Unlike the SHIME^®^ system, which utilizes peristaltic pumps to connect its various compartments (Van de Wiele et al. 2015), the MyBioScope model employs independent, modular mini-bioreactors. This design offers superior flexibility, allowing for one to four units to operate simultaneously—with the capacity for expansion—without the limitations of a linear, interconnected system. Furthermore, through precise automated control of pH, MyBioScope enables the targeted, simultaneous simulation of individual colonic regions or different parts of the GI tract. This autonomy allows researchers to apply distinct, customizable experimental conditions to each unit in parallel, significantly increasing throughput and experimental design capacity. Lastly, a principal benefit of the MyBioScope system is its flexibility to accurately replicate and modulate human gut microbiomes from diverse populations, maintaining distinct compositions from both healthy and disease-associated donors throughout the experimental duration. This stability ensures that the results of diverse interventions—such as supplementation—remain biologically valid and are not confounded by system-induced fluctuations.

## Conclusion

In conclusion, our novel MyBioScope model was optimized for an in vitro representation of the human gut microbiome using the DASbox^®^ mini bioreactor system and human stool samples. Our approach rapidly stabilizes the microbiome, maintains its composition over time, and is adaptable for studying disease scenarios or drug-microbiome interactions. The MyBioScope model exhibits great potential for in-depth exploring disease-specific microbiomes, offering valuable insights for integrating into fundamental studies and clinical applications.

## Supplementary Information

Supplementary Material 1.

## Data Availability

Raw sequence data are deposited in the National Center for Biotechnology Information (NCBI) sequence read archive (accession no. PRJNA1195243, PRJNA1191805, and PRJNA1197440).

## Abbreviations

GI: Gastrointestinal
LEfSe: Linear discriminant analysis effect size
NMR: Nuclear magnetic resonance
PCoA: Principal coordinates analysis
PCR: Polymerase chain reaction
PD: Phylogenetic diversity
PPI: Proton pump inhibitor
qPCR: Quantitative polymerase chain reaction
RDA: Redundancy analysis
SE: Standard error
SQ: Starting quantities
