# Supplementary material for: MyBioScope: a new frontier in gut microbiome and health research
Source: Bioresour Bioprocess. 2026 May 23;13(1):73. doi: 10.1186/s40643-026-01044-1 (PMC13198590; doi:10.1186/s40643-026-01044-1)
Supplement: Supplementary file 1 — Supplementary Material 1. [file 40643_2026_1044_MOESM1_ESM.docx]

*Supplementary Materials*

**MyBioScope: A New Frontier in Gut Microbiome and Health Research**

Kristina Žukauskaitė^1,2†^, Angela Horvath^1,2†^, Selina Tripolt^2,3^, Hansjörg Habisch^4^, Tobias Madl^4,5^, Christian Pacher-Deutsch^1,2^, Maximilian Nepel^1^, Irina Balazs^1,2^, Vanessa Stadlbauer^1,2,5*^

^1^ Division for Gastroenterology and Hepatology, Department of Internal Medicine, Medical University of Graz, Graz, Austria.

^2^ Division Translational Precision Medicine, Center for Biomarker Research in Medicine (CBmed GmbH), Graz, Austria.

^3^ Division of Medical Psychology, Psychosomatics and Psychotherapeutic Medicine, Medical University of Graz, Graz, Austria.

^4^ Otto Loewi Research Center, Division Medicinal Chemistry, Medical University of Graz, Graz, Austria.

^5^ BioTechMed-Graz, Graz, Austria.

*Corresponding author:

Prof. Vanessa Stadlbauer, MD, PhD

Address: Auenbruggerplatz 15, 8036 Graz, Austria

Tel.: 0043 316 385 82282

*E-mail: vanessa.stadlbauer@medunigraz.at*

†These authors contributed equally to this work.

**SUPPLEMENTARY FIGURES**


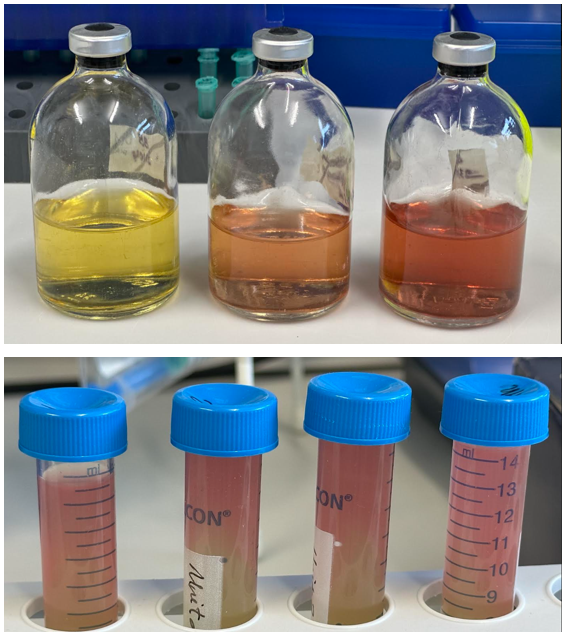


**Fig. S1.** The prepared Bryant and Burkey’s fermentation medium's anaerobic state was indicated visually through colour changes: in the upper picture, yellow indicates an anaerobic medium, orange indicates slight oxygen contamination, and red indicates oxygen contamination. The bottom picture illustrates the medium's colour shift upon exposure to oxygen after the sampling procedure, showcasing the rapid reaction time of the indicator resazurin.


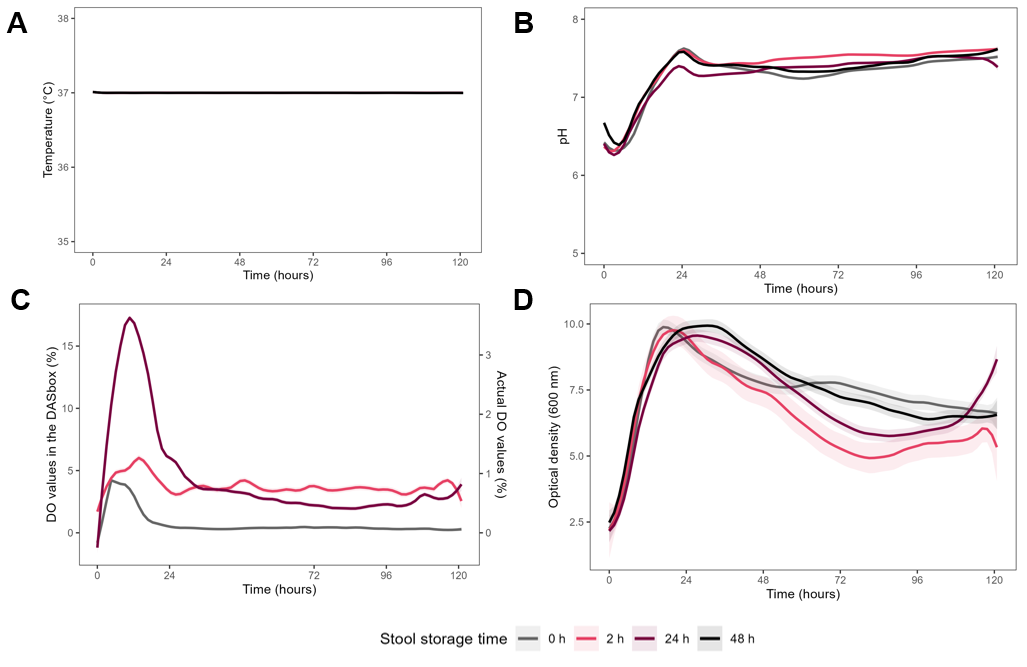


**Fig. S2.** Logged process parameters: (**A**) temperature, (**B**) pH, (**C**) dissolved oxygen (DO) level, and (**D**) optical density in the DASbox^®^ mini bioreactor system before and after supplementation with tested products. Colours represent four different tested stool storage times. Graph **C** depicts only three groups, as the fourth DO sensor broke during the experiment. The oxygen-sensitive indicator in the fermentation medium reassured no oxygenation.


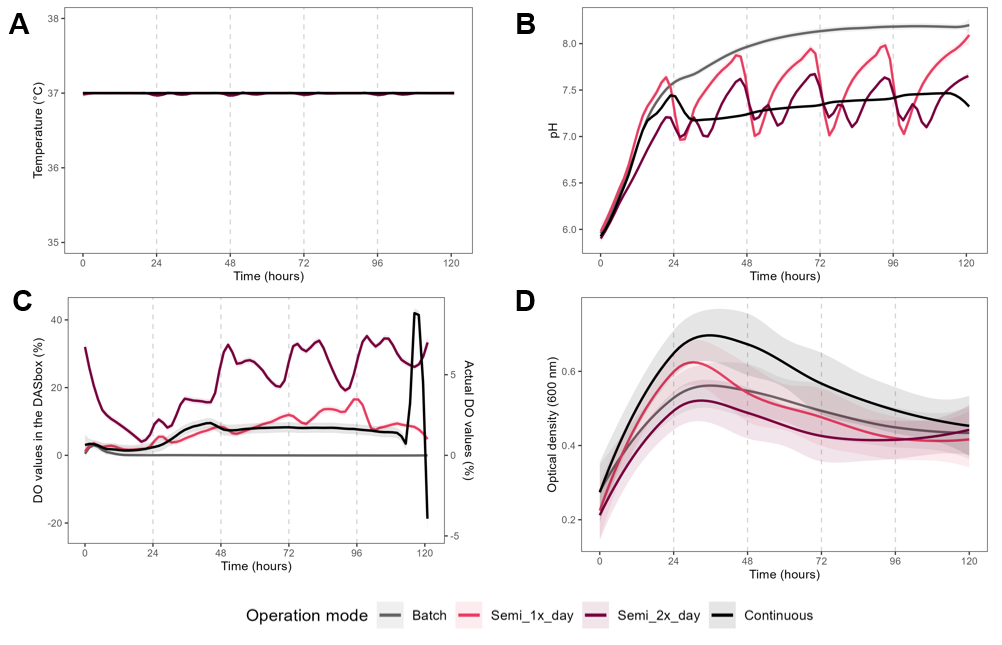


**Fig. S3.** Changes in process parameters: (**A**) temperature, (**B**) pH, (**C**) dissolved oxygen (DO) level and (**D**) optical density in the DASbox^®^ mini bioreactor system in different operational feeding (medium exchange) modes. Colours represent four tested modes: batch, semi-continuous (once and twice a day), and continuous. Please note that, graph **D** represents OD values measured using a plate reader, as two out of four OD sensors in the DASbox^®^ mini bioreactor system broke during the experimental setup.


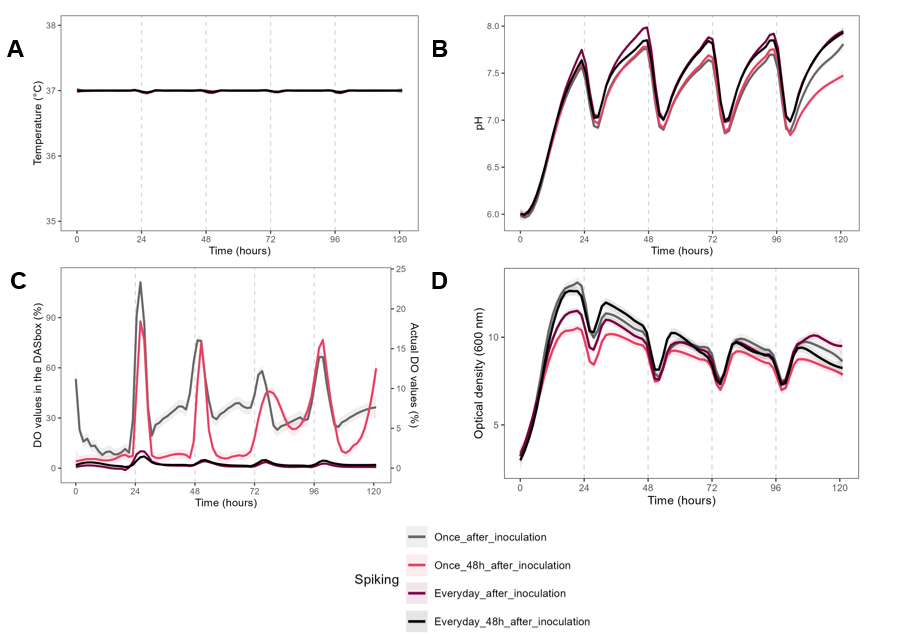


**Fig. S4**. Changes in process parameters: (**A**) temperature, (**B**) pH, (**C**) dissolved oxygen (DO) level, (**D**) optical density in the DASbox^®^ mini bioreactor system. The colours represent tested spiking conditions of oral bacteria, dashed lines indicate semi-continuous medium exchange.


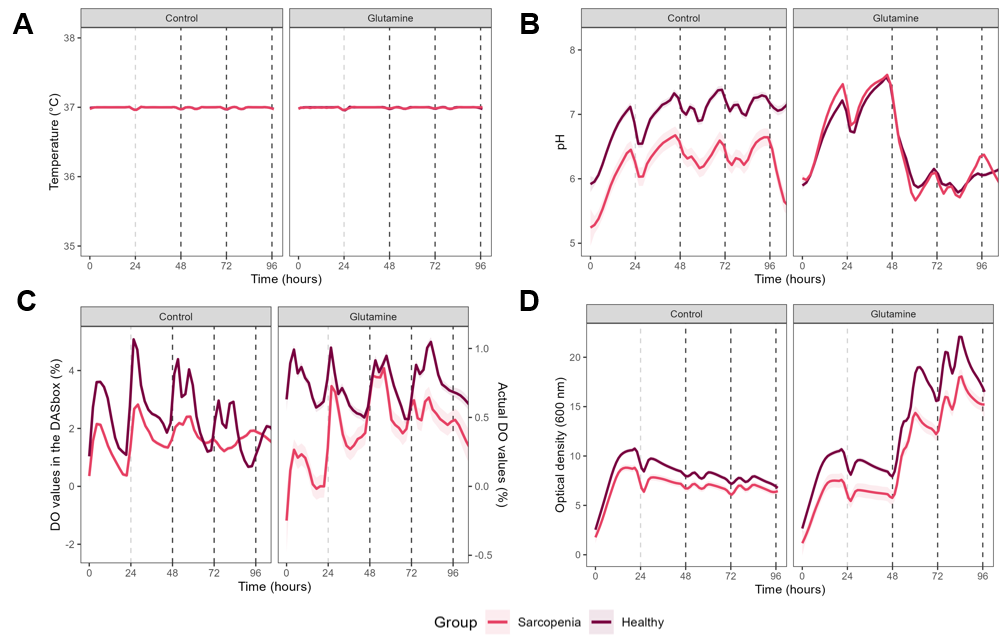


**Fig. S5.** Changes in process parameters: (**A**) temperature, (**B**) pH, (**C**) dissolved oxygen levels, and (**D**) optical density when supplementing glutamine after 48 h to two of four DASbox^®^ mini bioreactors. Colours represent two different tested groups: patients with sarcopenia and healthy controls.


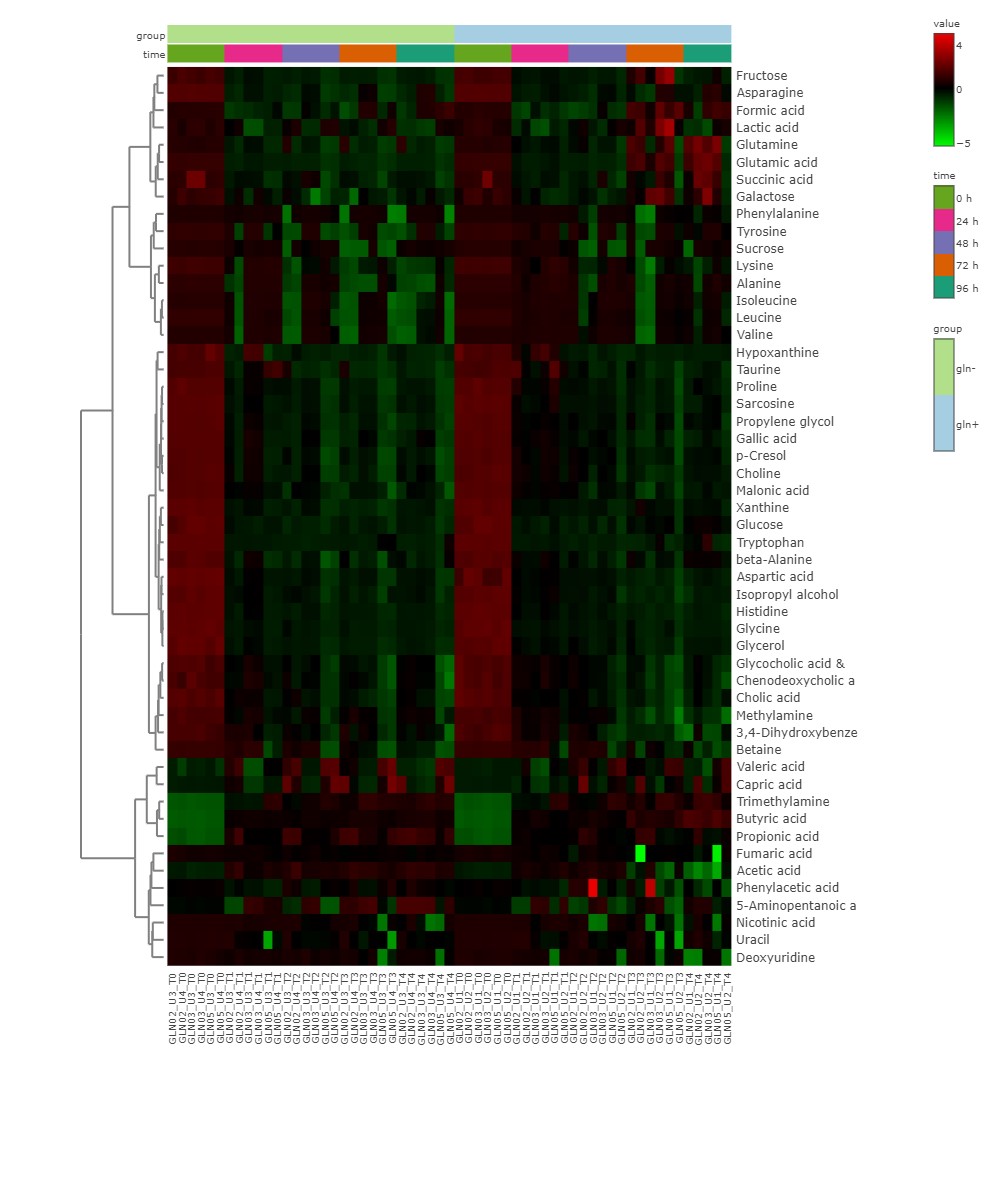


**Fig. S6.** Metabolic changes in sarcopenia-specific microbiomes cultivated in bioreactors and with glutamine supplementation (**gln+**) after 48 h post-inoculation or without (**gln-**).

**SUPPLEMENTARY TABLES**

**Table S1.** The composition of Bryant and Burkey medium used for anaerobic fermentation.

| **Reagent** | **Company** | **Cat. Number** | **Concentration (g/L)** |
| --- | --- | --- | --- |
| Peptone from caseine, tryptic digest | SIGMA-ALDRICH | 70172 | 15 |
| Yeast Extract, for use in microbial growth medium | SIGMA-ALDRICH | Y1625 | 5 |
| Meat extract | SIGMA-ALDRICH | 70164 | 7.5 |
| Sodium acetate | ROTH | X891.1 | 5 |
| L-cysteine hydrochloride | SIGMA-ALDRICH | C1276 | 0.5 |
| Resazurin (0.07 g in 70 mL distilled water) | SIGMA-ALDRICH |  | 800 µL |
| Distilled water |  |  | To 1 L |

**Table S2.** Technical details of setup of the *in vitro* human gut microbiome model, based on the DASbox^®^ mini bioreactor system.

| **Devices** | **Company** | **Cat. Number** |
| --- | --- | --- |
| *Base of the System* | | |
| DASbox^®^ Mini Bioreactor System MP8-pHpO | Eppendorf | - |
| Process Computer for Small-Scale Systems | Eppendorf | 76DGPCS |
| Uninterruptible Power Supply | Eppendorf | 76DGUPSU1 |
| DASbox® Vessel (with 2 Rushton-type impellers) | Eppendorf | 76SR0250ODLS |
| DASbox^®^ Feed Line Set | Eppendorf | 76DXFL05C11 |
| DASbox^®^ Overhead Drive | Eppendorf | 78525185 |
| DASbox^®^ Autoclavable Carrier | Eppendorf | 76DXBKT4 |
| *Sensors to Monitor Bioprocessess* | | |
| OxyFerm FDA 120 (DO sensor) | Hamilton | 237450 |
| VisiFerm DO ECS 120 (DO sensor) | Hamilton | 243666-211 |
| Dencytee 120 (OD sensor) | Hamilton | 243750 |
| EasyFerm Plus, PHI K8 120 (pH sensor) | Hamilton | 238633-1513 |
| Platinum RTD Temperature Sensor | Eppendorf | 78103304 |
| Level Sensor | Eppendorf | 78103145 |
| *Connecting Parts for the Sensors* | | |
| DO Sensor Cable | Eppendorf | 78522042 |
| Cell Density Combox | Hamilton | 243810 |
| Dencytee Pre-Amp | Hamilton | 243755 |
| Cables for Dencytee sensors | Hamilton | - |
| pH Sensor Cable | Eppendorf | 78522020 |
| Level Sensor Cable | Eppendorf | 78522031 |
| *Additional Exchangable Parts* | | |
| Sampling Port Systems | Eppendorf | 78510145 |
| Gassing Port Systems | Eppendorf | 78532034 |
| Medium Exchange Systems | Eppendorf | 78532036 |
| Tubing Clamp | Eppendorf | 78200119 |
| Rushton-Type Impeller | Eppendorf | 78107304 |

**Table S3.** List of all reagents used in this study.

| **Reagents** | **Company** | **Cat. Number** |
| --- | --- | --- |
| Bryant and Burkey medium suitable for microbiology, NutriSelect^®^ Plus | Merck | 91903 |
| Peptone from caseine, tryptic digest | SIGMA-ALDRICH | 70172 |
| Yeast Extract, for use in microbial growth medium | SIGMA-ALDRICH | Y1625 |
| Meat extract | SIGMA-ALDRICH | 70164 |
| Sodium acetate | ROTH | X891.1 |
| L-cysteine hydrochloride | SIGMA-ALDRICH | C1276 |
| Resazurin sodium salt | SIGMA-ALDRICH | R7017 |
| Potassium carbonate, 1 kg ≥98 % | ROTH | 7956.1 |
| Hydrochloric acid (2 N) | ROTH | T134.1 |
| Tryptic soy broth | SIGMA-ALDRICH | T8907 |
| L(+)-Lactic acid calcium salt pentahydrate | ROTH | 4071.1 |
| TWEEN^®^ 80, Viscous liquid | SIGMA-ALDRICH | P1754 |
| D-(+)-Glucose ≥99.5% | SIGMA-ALDRICH | G8270 |
| Putrescin, ≥99 % | ROTH | 8379.1 |
| Sodium thioglycolate | SIGMA | T0632 |
| Ethanol absolut | Merck | 1.00983.5000 |
| Sodium sulfate | ROTH | 8560.1 |
| QUANTOM^™^ Total Cell Staining Kit | Logos Biosystems | Q13501 |
| Tris | ROTH | 5429.1 |
| EDTA | ROTH | 8043.1 |
| Triton^®^ X-100 | ROTH | 3051.3 |
| Lysozym | ROTH | 8259.3 |
| DNeasy Blood & Tissue Kit | QIAGEN | 69506 |
| GoTaq^®^ qPCR Master Mix | Promega GmbH | A6002 |
| Target Specific PCR Primers | Eurofins Genomics | - |
| Nuclease-Free Water | Promega | P119E |
| Kabi Glutamine Powder | Fresenius Kabi Deutschland GmbH | - |
| ALPHAGAZ^TM^ 1 N_2_ | Air Liquide | - |
| Gas mixture:  CO_2_ N45  H N30  Rest N50 | Air Liquide | - |

**Table S4.** List of the materials and devices used in this study.

| **Materials and Devices** | **Company** | **Cat. Number** |
| --- | --- | --- |
| GutAlive^®^ Anaerobic microbiome collection kit | MicroViable Therapeutics | - |
| Thermo Heraeus Multifuge 3 L-R Refrigerated Centrifuge | Thermo Heraeus Kendro | 2962 |
| Thermo Scientific™ Fresco™ 17 Microcentrifuge | Fisher Scientific | 75002402 |
| QUANTOM Tx™ Microbial Cell Counter | Logos Biosystems | Q10001 |
| QUANTOM™ Centrifuge | Logos Biosystems | Q10002 |
| QUANTOM™ M50 Cell Counting Slides | Logos Biosystems | Q12001 |
| RH basic 2 Magnetic Stirrer/Hotplate | IKA | 0003339000 |
| RCT standard Magnetic Stirrer/Hotplate | IKA | 0003622000 |
| MS 3 Basic Vortex Mixer | IKA | 0003617000 |
| New Brunswick™ Excella^®^ E24/E24R Shaker | Eppendorf | Discontinued |
| Grant Bio PCV-2400 Combined Centrifuge and Vortex Mixer | Grant Instruments | 11441028 |
| Whitley A85 anaerobic workstation | Don Whitley Scientific | - |
| 15 ml gas glass tubes with gassing cap | - | - |
| 100 ml glass flasks | - | - |
| Disposable cuvettes | BRAND GmbH | 7590 |
| Aluminum crimp caps, 20 mm | Ochs Glasgeratebau | 102050 |
| Manual decapper for 20 mm aluminum crimp caps | LLG LABWARE | 9.003 369 |
| Manual crimper, height adjustable, for 20 mm aluminum crimp caps | LLG LABWARE | 9.003 475 |
| 15 mL Conical Polypropylene Centrifuge Tube with Dome Seal Screw Cap | Corning | 352097 |
| 50 mL Polypropylene Centrifuge Tube with Flat Screw Cap | Corning | 352098 |
| SmartBoats™ weighing boats | LevGo | 20202 |
| Nalgene^®^ Syringe Filter with SFCA Cellulose Acetate Membrane, Sterile, Diameter: 25mm, Pore Size: 0.2µm | Thermo Scientific | 190-2520 |
| Nalgene^®^ Syringe Filter, Sterile SFCA membrane, Diameter: 25mm, Pore Size: 0.2µm | Thermo Scientific | 723-2520 |
| VACUETTE^®^ Luer Adapter 20G sterile | Manufacturer: Nipro Medical Industries Ltd.;  Imported by: Greiner bio-one | 450070 |
| Sterican Disposable Injection Needle 0.6x30mm 23G | Braun | 4657640 |
| Luer-Lok™ 50-mL syringe | BD | 300865 |
| 2-part disposable syringes 20 mL | CHIRANA | CH020L |
| Injekt^®^ Solo Syringe (Luer) 10 ml | Braun | 4606108V |
| 2-part disposable syringes 5 mL | CHIRANA | CH002L |
| Luer-Lok™ 1-mL syringe | BD | 309628 |
| CFX96 Touch Real-Time PCR Detection System | BIO-RAD | 1855196 (soon to be discontinued) |
| Hard-Shell PCR Plates 96-well, thin-wall | BIO-RAD | HSP9655 |
| Microseal^®^ 'B' PCR Plate Sealing Film, adhesive, optical | BIO-RAD | MSB1001 |
| Glass pellets | Assistant | 41401001 |
| 1.5 mL SC Micro Tube PCR-PT | SARSTEDT | 8082711 |
| NanoDrop™ 2000/2000c Spectrophotometer | Fisher Scientific | ND2000 |
| PARAFILM^®^ Sealing film | CARL-ROTH | CNP8.1 |

**Table S5.** The composition of oral bacteria fermentation medium of *Veillonella parvula* and *Streptococcus salivarius*.

| **Reagent** | **Company** | **Cat. Number** | **Concentration (g/L)** |
| --- | --- | --- | --- |
| ***Veillonella parvula*** | | | |
| Peptone from caseine, tryptic digest | SIGMA-ALDRICH | 70172 | 5 |
| Yeast Extract, for use in microbial growth medium | SIGMA-ALDRICH | Y1625 | 3 |
| L(+)-Lactic acid calcium salt pentahydrate | ROTH | 4071.1 | 7.5 |
| Sodium thioglycolate | SIGMA | T0632 | 0.75 |
| TWEEN^®^ 80, Viscous liquid | SIGMA-ALDRICH | P1754 | 1 |
| D-(+)-Glucose ≥99.5% | SIGMA-ALDRICH | G8270 | 1 |
| Putrescin, ≥99 % | ROTH | 8379.1 | 0.003 |
| Resazurin sodium salt | SIGMA-ALDRICH | R7017 | 0.001 |
| Distilled water | - | | To 1 L |
| Potassium carbonate, 1 kg ≥98 % | ROTH | 7956.1 | - |
| ***Streptococcus salivarius* (M92 medium)** | | | |
| Tryptic soy broth | SIGMA-ALDRICH | T8907 | 30 |
| Yeast Extract, for use in microbial growth medium | SIGMA-ALDRICH | Y1625 | 3 |
| Distilled water | - | | To 1 L |
| Hydrochloric acid (2 N) | ROTH | T134.1 | - |

**Table S6.** The composition of lysis buffer used for DNA extraction.

| **Reagent** | **Company** | **Cat. Number** | **Concentration** |
| --- | --- | --- | --- |
| Hydrochloric acid (2 N) | ROTH | T134.1 | 20 mM Tris·Cl,  pH = 8.0 |
| TRIS | ROTH | 5429.1 |  |
| EDTA | ROTH | 8043.1 | 2 mM |
| Triton^®^ X-100 | ROTH | 3051.3 | 1.2% |
| Lysozym | ROTH | 8259.3 | 20 mg/mL |
| Nuclease-Free Water | Promega | P119E | - |

**Table S7.** Sequences of primers used for the testing of oral bacteria abundance in the bioreactors and data normalization.

|  | **PMID** |  | **Primer sequence** | **Amplicon size, bp** |
| --- | --- | --- | --- | --- |
| *Veillonella parvula* | 27326455 | F | GTAACAAAGGTGTCGTTTCTCG | 311 |
|  |  | R | CGTAACATCTTCCGAAACTTTC |  |
| *Streptococcus salivarius* | 30022122 | F | TGAACAAGCRGTWGTCGGTAAC | 108 |
|  |  | R | ACTCCGTGTCCAACCAAATC |  |
| *16S rRNA* gene F1048/R1194 | - | F | GTGSTGCAYGGYTGTCGTCA | 146 |
|  |  | R | ACGTCRTCCMCACCTTCCTC |  |

**Table S8.** Components of the quantitative polymerase chain reaction for the testing of oral bacteria abundance in the bioreactors and data normalization.

| **Reaction component** | **Final concentration of reaction components**  **per well** | | |
| --- | --- | --- | --- |
|  | ***V. parvula*** | ***S. salivarius*** | ***16S rRNA* gene** |
| GoTaq^®^ qPCR Master Mix | 0.75x | 0.5x | 0.7x |
| Primer Mix (F + R) | 0.05 µM | 0.05 µM | 0.05 µM |
| Nuclease Free Water | - | - | - |
| DNA sample | 0.25 ng/µL | 0.5 ng/µL | 0.25 ng/µL |
| **Final volume (µL) per well** | **10** | **10** | **10** |

**Table S9.** Temperature regimes used for quantitative polymerase chain reaction for the testing of oral bacteria abundance in the bioreactors and data normalization.

| **Process** | **Temperature (°C)** | **Time** | **Cycles** |
| --- | --- | --- | --- |
| ***Streptococcus salivarius*** | | | |
| UDG Activation | 50 | 2 min | - |
| DNA polymerase activation | 95 | 10 min | - |
| Denaturation | 95 | 15 sec | 35 |
| Annealing/Extension | 60 | 1 min |  |
| Melting Curve | 55 °C to 95 °C | Increment 1 °C | - |
| ***Veillonella parvula*** | | | |
| DNA polymerase activation | 95 | 3 min | - |
| Denaturation | 95 | 30 sec | 36 |
| Annealing | 53 | 30 sec |  |
| Extension | 72 | 1 min 30 sec |  |
| Melting Curve | 55 °C to 95 °C | Increment 0.5 °C | - |
| ***16S rRNA* gene** | | | |
| DNA polymerase activation | 95 | 10 min | - |
| Denaturation | 95 | 15 sec | 39 |
| Annealing | 60 | 1 min |  |
| Melting Curve | 60 °C to 95 °C | Increment 1 °C | - |

**Table S10.** Summary of the technical details of the study.

| Part of the Study | Donors | Age | Volume of the Bioreactor | Concentration of Inoculum | Feeding Mode | Length of Cultivation | Fermentation Medium | Medium Exchange |
| --- | --- | --- | --- | --- | --- | --- | --- | --- |
| Microbiome preservation (GutAlive^®^) | 3 healthy females | 25-30 (avg. 27.7 ± 5 years) | 120 mL | 10% | Continuous | 120 h | Bryant and Burkey (made from separate components in the laboratory) | ~3 mL/rate |
| Feeding modes | 2 healthy females and 1 healthy male | 26-31  (avg. 28.7 ± 5 years) | 200 mL | 20% | Continuous, semi-continuous (once and twice daily), batch | 120 h | Bryant and Burkey (commercially available) | Cont.: ~3 mL/rate  Semi-Cont.: 25% or 50%  Batch: - |
| Disease modelling (Sarcopenia) | 3 sarcopenia patients (2 males and 1 female)  3 healthy donors (two males and one female) | Sarcopenia group: 25-70 years, avg. 49.0 ± 22.6 years  Control group: 28-32 years, avg. 29.3 ± 2.3 years | 200 mL | 20% | Semi-continuous (twice daily) | 96 h | Bryant and Burkey (commercially available) | 25% twice daily |
| PPI-induced oralization model | 3 healthy females | 25-30 (avg. 27.7 ± 5 years) | 200 mL | 20% | Semi-continuous (once daily) | 120 h | Bryant and Burkey (commercially available) | 50% once daily |

**Table S11.** Summary of the sequencing reads after filtering, and rarefaction depth for the stool and bacterial pellet samples used in this study.

| **Part of the study** | **Truncating length** | **The number of sequencing reads** | | | | **Chosen rarefaction depth** | **Samples removed after rarefaction** |
| --- | --- | --- | --- | --- | --- | --- | --- |
|  |  | **Total** | **Average** | **Minimum** | **Maximum** |  |  |
| Microbiome preservation (GutAlive^®^) | F-250  R-200 | 1,588,387 | 33,091 | 13,302 | 74,263 | **13,302** | 0 |
| Feeding modes | F-280  R-250 | 11,487,131 | 147,270 | 49,554 | 480,337 | **49,554** (if all samples are included);  **69,513** (if 3 stool, 3 slurry samples not included) | |
| Disease modelling (Sarcopenia) | F-280  R-220 | 2,117,784 | 21,178 | 13,862 | 29,259 | **13,862** (if all samples are included);  **14,620** (if six stool samples are removed) | 2 |

**Table S12.** Linear mixed-effects model results for alpha-diversity parameters. The table presents the estimates, standard errors (S.E.), t-values (t val.), degrees of freedom (d.f.), and p-values for five alpha diversity indices: Richness, Shannon, Inverse Simpson, Evenness, and Phylogenetic Diversity (PD).

| **Source of variation** | **Est.** | **S.E.** | ***t* val.** | **d.f.** | ***p*-value** |
| --- | --- | --- | --- | --- | --- |
| *Richness index* | | | | | |
| Stool Storage Time | -0.591 | 0.605 | -0.978 | 32.000 | 0.336 |
| Cultivation Time | -2.069 | 0.262 | -7.896 | 32.000 | **0.000** |
| Stool Storage Time * Cultivation Time | 0.007 | 0.010 | 0.692 | 32.000 | 0.494 |
| *Shannon index* | | | | | |
| Stool Storage Time | -0.002 | 0.003 | -0.721 | 32.000 | 0.476 |
| Cultivation Time | -0.010 | 0.001 | -6.881 | 32.000 | **0.000** |
| Stool Storage Time * Cultivation Time | 0.000 | 0.000 | 0.192 | 32.000 | 0.849 |
| *Inverse Simpson index* | | | | | |
| Stool Storage Time | -0.178 | 0.162 | -1.101 | 32.000 | 0.279 |
| Cultivation Time | -0.358 | 0.070 | -5.105 | 32.000 | **0.000** |
| Stool Storage Time * Cultivation Time | 0.002 | 0.003 | 0.579 | 32.000 | 0.566 |
| *Evenness index* | | | | | |
| Stool Storage Time | -0.000 | 0.000 | -0.432 | 32.000 | 0.669 |
| Cultivation Time | -0.001 | 0.000 | -3.457 | 32.000 | **0.002** |
| Stool Storage Time * Cultivation Time | -0.000 | 0.000 | -0.233 | 32.000 | 0.817 |
| *PD – whole tree* | | | | | |
| Stool Storage Time | -0.011 | 0.040 | -0.282 | 30.000 | 0.780 |
| Cultivation Time | -0.114 | 0.017 | -6.591 | 30.000 | **0.000** |
| Stool Storage Time * Cultivation Time | 0.000 | 0.001 | 0.053 | 30.000 | 0.958 |

**Table S13.** Results of PERMANOVA analysis. This table presents the PERMANOVA analysis results, including the proportion of explained variation (R^2^), statistical significance (*p*-values), and the F value for each factor. The F value represents the ratio of between-group variation to within-group variation and assesses the significance of the factor's effect.

| **Source of variation** | | **Df** | **Sum of sq.** | **R^2^** | **F** | ***p*-value** |
| --- | --- | --- | --- | --- | --- | --- |
| *PCoA based on unifrac* | | | | | | |
| Stool Storage Time | | 3 | 0.3297 | 0.02784 | 0.4084 | 0.843 |
| Cultivation Time | | 3 | 2.2551 | 0.19045 | 2.7937 | **0.001** |
| Stool Storage Time* Cultivation Time | | 9 | 0.6460 | 0.05456 | 0.2668 | 1.000 |
| *PCoA based on wunifrac* | | | | | | |
| Stool Storage Time | | 3 | 0.00625 | 0.01406 | 0.2276 | 0.983 |
| Cultivation Time | | 3 | 0.13094 | 0.29469 | 4.7718 | **0.001** |
| Stool Storage Time* Cultivation Time | | 9 | 0.01445 | 0.03253 | 0.1756 | 1.000 |
| *PCoA based on bray* | | | | | | |
| Stool Storage Time | | 3 | 0.3546 | 0.02254 | 0.3373 | 0.846 |
| Cultivation Time | | 3 | 3.6496 | 0.23198 | 3.4712 | **0.001** |
| Stool Storage Time* Cultivation Time | | 9 | 0.5137 | 0.03265 | 0.1629 | 1.000 |
| *PCoA based on jaccard* | | | | | | |
| Stool Storage Time | 3 | | 0.5840 | 0.03200 | 0.4695 | 0.847 |
| Cultivation Time | 3 | | 3.3681 | 0.18455 | 2.7077 | **0.001** |
| Stool Storage Time* Cultivation Time | 9 | | 1.0301 | 0.05644 | 0.2761 | 1.000 |

**Table S14.** Linear mixed-effects model results for alpha-diversity parameters. The table presents the estimates, standard errors (S.E.), t-values (t val.), degrees of freedom (d.f.), and p-values for four alpha diversity indices: Richness, Shannon, Inverse Simpson, and Evenness. Only collected samples from the DASbox^®^ were included in this analysis (3 stool samples and 3 slurry samples were excluded).

| **Source of variation** | **Est.** | **S.E.** | ***t* val.** | **d.f.** | ***p*-value** |
| --- | --- | --- | --- | --- | --- |
| *Richness index* | | | | | |
| Continuous | -64.143 | 53.749 | -1.193 | 62.000 | 0.237 |
| Semi_continuous_1x | -79.175 | 53.749 | -1.473 | 62.000 | 0.146 |
| Semi_continuous_2x | -92.603 | 53.749 | -1.723 | 62.000 | 0.090 |
| Cultivation Time | -1.801 | 0.523 | -3.444 | 62.000 | **0.001** |
| Cultivation Time * Continuous | -1.195 | 0.740 | -1.615 | 62.000 | 0.111 |
| Cultivation Time * Semi_continuous_1x | -0.148 | 0.740 | -0.200 | 62.000 | 0.842 |
| Cultivation Time * Semi_continuous_2x | -0.633 | 0.740 | -0.855 | 62.000 | 0.396 |
| *Shannon index* | | | | | |
| Continuous | 0.034 | 0.102 | 0.331 | 62.000 | 0.742 |
| Semi_continuous_1x | -0.048 | 0.102 | -0.475 | 62.000 | 0.636 |
| Semi_continuous_2x | 0.014 | 0.102 | 0.142 | 62.000 | 0.887 |
| Cultivation Time | -0.003 | 0.001 | -3.111 | 62.000 | **0.003** |
| Cultivation Time * Continuous | -0.006 | 0.001 | -4.364 | 62.000 | **0.000** |
| Cultivation Time * Semi_continuous_1x | -0.002 | 0.001 | -1.338 | 62.000 | 0.186 |
| Cultivation Time * Semi_continuous_2x | -0.005 | 0.001 | -3.503 | 62.000 | **0.001** |
| *Inverse Simpson index* | | | | | |
| Continuous | -8.879 | 8.354 | -1.063 | 62.000 | 0.292 |
| Semi_continuous_1x | -11.034 | 8.354 | -1.321 | 62.000 | 0.191 |
| Semi_continuous_2x | 1.625 | 8.354 | 0.195 | 62.000 | 0.846 |
| Cultivation Time | -0.211 | 0.081 | -2.598 | 62.000 | **0.012** |
| Cultivation Time * Continuous | -0.089 | 0.115 | -0.778 | 62.000 | 0.439 |
| Cultivation Time * Semi_continuous_1x | -0.003 | 0.115 | -0.023 | 62.000 | 0.982 |
| Cultivation Time * Semi_continuous_2x | -0.185 | 0.115 | -1.608 | 62.000 | 0.113 |
| *Evenness index* | | | | | |
| Continuous | 0.006 | 0.014 | 0.398 | 62.000 | 0.692 |
| Semi_continuous_1x | -0.004 | 0.014 | -0.268 | 62.000 | 0.790 |
| Semi_continuous_2x | 0.010 | 0.014 | 0.712 | 62.000 | 0.479 |
| Cultivation Time | -0.000 | 0.000 | -1.638 | 62.000 | 0.106 |
| Cultivation Time * Continuous | -0.000 | 0.000 | -2.391 | 62.000 | **0.020** |
| Cultivation Time * Semi_continuous_1x | -0.000 | 0.000 | -0.432 | 62.000 | 0.667 |
| Cultivation Time * Semi_continuous_2x | -0.000 | 0.000 | -2.218 | 62.000 | **0.030** |
| *PD – whole tree* | | | | | |
| Continuous | -0.729 | 0.973 | -0.749 | 62.000 | 0.457 |
| Semi_continuous_1x | -0.840 | 0.973 | -0.863 | 62.000 | 0.392 |
| Semi_continuous_2x | -1.141 | 0.973 | -1.173 | 62.000 | 0.245 |
| Cultivation Time | -0.026 | 0.009 | -2.778 | 62.000 | **0.007** |
| Cultivation Time * Continuous | -0.027 | 0.013 | -2.028 | 62.000 | **0.047** |
| Cultivation Time * Semi_continuous_1x | -0.005 | 0.013 | -0.372 | 62.000 | 0.711 |
| Cultivation Time * Semi_continuous_2x | -0.020 | 0.013 | -1.470 | 62.000 | 0.147 |

**Table S15.** PERMANOVA analysis results of microbiome composition changes throughout the treatment of experimental time, using different feeding modes.

| **Source of variation** | | **Df** | **Sum of sq.** | **R^2^** | **F** | ***p*-value** |
| --- | --- | --- | --- | --- | --- | --- |
| *PCoA based on unifrac* | | | | | | |
| Timepoint | | 1 | 1.0604 | 0.05466 | 3.9784 | **0.001** |
| Feeding Mode | | 3 | 0.7681 | 0.03959 | 0.9606 | **0.001** |
| Timepoint* Feeding Mode | | 3 | 0.5140 | 0.02649 | 0.6428 | **0.009** |
| *PCoA based on wunifrac* | | | | | | |
| Timepoint | | 1 | 0.002538 | 0.07863 | 6.1087 | **0.001** |
| Feeding Mode | | 3 | 0.001883 | 0.05833 | 1.5105 | **0.001** |
| Timepoint* Feeding Mode | | 3 | 0.001265 | 0.03919 | 1.0150 | **0.003** |
| *PCoA based on bray* | | | | | | |
| Timepoint | | 1 | 1.7435 | 0.07391 | 5.6250 | **0.001** |
| Feeding Mode | | 3 | 1.2741 | 0.05401 | 1.3702 | **0.001** |
| Timepoint* Feeding Mode | | 3 | 0.7338 | 0.03111 | 0.7891 | **0.019** |
| *PCoA based on jaccard* | | | | | | |
| Timepoint | 1 | | 1.5969 | 0.05838 | 4.3942 | **0.001** |
| Feeding Mode | 3 | | 1.5652 | 0.05722 | 1.4357 | **0.001** |
| Timepoint* Feeding Mode | 3 | | 0.9349 | 0.03418 | 0.8575 | **0.018** |

**Table S16.** Linear mixed-effects model results for oral bacteria abundance. The table presents the estimates, standard errors (S.E.), t-values (t val.), degrees of freedom (d.f.), and *p*-values for correlations with the abundance of oral bacteria. Timepoints 0 and 24 hours are excluded, as two of four supplementations started after 48 hours post-inoculation.

|  | **Source of variation** | **Est.** | **S.E.** | **t val.** | **d.f.** | ***p*-value** |
| --- | --- | --- | --- | --- | --- | --- |
| *Veillonella parvula* | | | | | | |
| 1 | Everyday_after_inoculation | 259.042 | 98.054 | 2.642 | 62.000 | **0.010** |
| 2 | Once_48h_after_inoculation | 39.194 | 98.054 | 0.400 | 62.000 | 0.691 |
| 3 | Once_after_inoculation | 220.473 | 98.054 | 2.248 | 62.000 | **0.028** |
| 4 | Cultivation Time | 1.567 | 0.954 | 1.642 | 62.000 | 0.106 |
| 5 | Everyday_after_inoculation* Cultivation Time | -0.652 | 1.349 | -0.483 | 62.000 | 0.631 |
| 6 | Once_48h_after_inoculation* Cultivation Time | -1.483 | 1.349 | -1.099 | 62.000 | 0.276 |
| 7 | Once_after_inoculation* Cultivation Time | -2.096 | 1.349 | -1.553 | 62.000 | 0.125 |
| *Streptococcus salivarius* | | | | | | |
| 1 | Everyday_after_inoculation | 220.005 | 151.617 | 1.451 | 62.000 | 0.152 |
| 2 | Once_48h_after_inoculation | 72.059 | 151.617 | 0.475 | 62.000 | 0.636 |
| 3 | Once_after_inoculation | 225.735 | 151.617 | 1.489 | 62.000 | 0.142 |
| 4 | Cultivation Time | 4.140 | 1.475 | 2.806 | 62.000 | **0.007** |
| 5 | Everyday_after_inoculation* Cultivation Time | 2.470 | 2.087 | 1.184 | 62.000 | 0.241 |
| 6 | Once_48h_after_inoculation* Cultivation Time | -1.836 | 2.087 | -0.880 | 62.000 | 0.382 |
| 7 | Once_after_inoculation* Cultivation Time | -5.231 | 2.087 | -2.507 | 62.000 | **0.015** |

**Table S17.** Linear mixed-effects model results for alpha-diversity parameters. The table presents the estimates, standard errors (S.E.), t-values (t val.), degrees of freedom (d.f.), and p-values for four alpha diversity indices: Richness, Shannon, Inverse Simpson, and Evenness.

| **Source of variation** | **Est.** | **S.E.** | ***t* val.** | **d.f.** | ***p*-value** |
| --- | --- | --- | --- | --- | --- |
| *Richness index* | | | | | |
| Healthy_Glutamine | 5.601 | 12.244 | 0.457 | 82.130 | 0.649 |
| Sarcopenia_Control | 14.827 | 17.960 | 0.826 | 10.779 | 0.427 |
| Sarcopenia_Glutamine | 17.389 | 17.960 | 0.968 | 10.779 | 0.354 |
| Timepoint | -0.072 | 0.136 | -0.532 | 82.119 | 0.596 |
| Healthy_Glutamine:Timepoint | -0.624 | 0.190 | -3.284 | 82.173 | **0.002** |
| Sarcopenia_Control:Timepoint | 0.001 | 0.187 | 0.003 | 82.071 | 0.998 |
| Sarcopenia_Glutamine:Timepoint | -0.395 | 0.187 | -2.115 | 82.071 | **0.037** |
| *Shannon index* | | | | | |
| Healthy_Glutamine | 0.034 | 0.148 | 0.227 | 82.159 | 0.821 |
| Sarcopenia_Control | -0.203 | 0.206 | -0.987 | 12.453 | 0.342 |
| Sarcopenia_Glutamine | -0.138 | 0.206 | -0.673 | 12.453 | 0.513 |
| Timepoint | 0.001 | 0.002 | 0.523 | 82.146 | 0.602 |
| Healthy_Glutamine:Timepoint | -0.005 | 0.002 | -2.073 | 82.211 | **0.041** |
| Sarcopenia_Control:Timepoint | 0.002 | 0.002 | 1.013 | 82.089 | 0.314 |
| Sarcopenia_Glutamine:Timepoint | -0.006 | 0.002 | -2.599 | 82.089 | **0.011** |
| *Inverse Simpson index* | | | | | |
| Healthy_Glutamine | 0.072 | 11.926 | 0.006 | 82.247 | 0.995 |
| Sarcopenia_Control | -16.102 | 14.788 | -1.089 | 18.834 | 0.290 |
| Sarcopenia_Glutamine | -16.049 | 14.788 | -1.085 | 18.834 | 0.292 |
| Timepoint | 0.152 | 0.132 | 1.154 | 82.226 | 0.252 |
| Healthy_Glutamine:Timepoint | -0.286 | 0.185 | -1.546 | 82.327 | 0.126 |
| Sarcopenia_Control:Timepoint | 0.116 | 0.182 | 0.637 | 82.139 | 0.526 |
| Sarcopenia_Glutamine:Timepoint | -0.336 | 0.182 | -1.846 | 82.139 | 0.068 |
| *Evenness index* | | | | | |
| Healthy_Glutamine | 0.002 | 0.023 | 0.104 | 82.234 | 0.917 |
| Sarcopenia_Control | -0.047 | 0.029 | -1.610 | 17.765 | 0.125 |
| Sarcopenia_Glutamine | -0.037 | 0.029 | -1.261 | 17.765 | 0.224 |
| Timepoint | 0.000 | 0.000 | 0.804 | 82.214 | 0.424 |
| Healthy_Glutamine:Timepoint | -0.000 | 0.000 | -1.161 | 82.309 | 0.249 |
| Sarcopenia_Control:Timepoint | 0.000 | 0.000 | 1.198 | 82.132 | 0.234 |
| Sarcopenia_Glutamine:Timepoint | -0.001 | 0.000 | -2.273 | 82.132 | **0.026** |
| *PD – whole tree* | | | | | |
| Healthy_Glutamine | 0.573 | 0.644 | 0.890 | 82.078 | 0.376 |
| Sarcopenia_Control | -0.645 | 1.115 | -0.579 | 7.756 | 0.579 |
| Sarcopenia_Glutamine | -0.567 | 1.115 | -0.509 | 7.756 | 0.625 |
| Timepoint | -0.004 | 0.007 | -0.514 | 82.071 | 0.609 |
| Healthy_Glutamine:Timepoint | -0.038 | 0.010 | -3.840 | 82.105 | **0.000** |
| Sarcopenia_Control:Timepoint | -0.004 | 0.010 | -0.372 | 82.043 | 0.711 |
| Sarcopenia_Glutamine:Timepoint | -0.025 | 0.010 | -2.566 | 82.043 | **0.012** |

**Table S18.** Results of PERMANOVA analysis. This table presents the PERMANOVA analysis results, including the proportion of explained variation (R^2^), statistical significance (*p*-values), and the F value for each factor. The F value represents the ratio of between-group variation to within-group variation and assesses the significance of the factor's effect.

| **Source of variation** | | **Df** | **Sum of sq.** | **R^2^** | **F** | ***p*-value** |
| --- | --- | --- | --- | --- | --- | --- |
| *PCoA based on unifrac* | | | | | | |
| Group | | 3 | 1.7409 | 0.12360 | 4.6821 | **0.001** |
| Timepoint | | 3 | 1.8740 | 0.13305 | 5.0400 | **0.001** |
| Group*Timepoint | | 9 | 0.8022 | 0.05695 | 0.7191 | **0.041** |
| *PCoA based on wunifrac* | | | | | | |
| Group | | 3 | 0.9261 | 0.14698 | 9.9146 | **0.001** |
| Timepoint | | 3 | 2.2609 | 0.35882 | 24.2048 | **0.001** |
| Group*Timepoint | | 9 | 0.6854 | 0.10877 | 2.4458 | **0.001** |
| *PCoA based on bray* | | | | | | |
| Group | | 3 | 4.190 | 0.12383 | 4.5575 | **0.001** |
| Timepoint | | 3 | 3.677 | 0.10867 | 3.9996 | **0.001** |
| Group*Timepoint | | 9 | 2.066 | 0.06105 | 0.7490 | **0.003** |
| *PCoA based on jaccard* | | | | | | |
| Group | 3 | | 3.943 | 0.10225 | 3.6326 | **0.001** |
| Timepoint | 3 | | 3.355 | 0.08700 | 3.0911 | **0.001** |
| Group*Timepoint | 9 | | 3.043 | 0.07892 | 0.9346 | **0.002** |

**SUPPLEMENTARY METHODS**

## Targeted Metabolomics by NMR spectroscopy

Proton nuclear magnetic resonance (1H-NMR) spectroscopy was employed to characterize the metabolic profiles of the sample supernatants. Metabolic phenotyping followed the protocol described by Reisinger et al. [1] and was carried out at the “Integrative Structural Biology and Metabolomics” laboratory, directed by Prof. Tobias Madl.

In brief, enzyme quenching was achieved by mixing the samples with methanol, followed by storage at −20 °C for 30 min. Supernatants were then collected by centrifugation, dried, and reconstituted in NMR buffer prepared in D_2_O. The samples were transferred into NMR tubes and analyzed at 37 °C using an AVANCE™ Neo Bruker Ultrashield 600 MHz spectrometer equipped with a TXI probe. Spectra were acquired using a 1D CPMG (Carr–Purcell–Meiboom–Gill) pulse sequence with water suppression and pre-saturation. Data acquisition and spectral processing were performed with Bruker TopSpin software (version 4.5) and MATLAB^®^ 2014b [1].

In total, 52 metabolites were quantified using a targeted metabolomics approach. For data analysis, reactor duplicate averages were calculated, and the data were stratified by disease status (sarcopenia *vs.* healthy), treatment (gln+, glutamine supplementation *vs*. gln-, no supplementation), and timepoint (pre-supplementation, T0–T2; post-supplementation, T3–T4). In this analysis, we focused on the sarcopenia group and evaluated the impact of glutamine supplementation on the microbiome. The data were analysed and visualized using MetaboAnalyst (version 6.0)[2].

## Amplicon Sequencing

- 1. **Microbiome Collection Optimization: Stool Sample Collection and Anaerobic Inoculum Preparation**

Bacterial DNA isolation and *16S rRNA* gene sequencing were performed by Institute for Clinical Molecular Biology at Kiel University, Kiel, Germany. Stool samples were thawed, and DNA was extracted using the QIAamp FAST DNA stool mini kit (QIAGEN, Hilden, Germany) automated using the QIAcube (QIAGEN, Hilden, Germany). Approximately 200 mg stool were transferred to 0.70 mm garnet bead tubes filled with 1 mL InhibitEx buffer (QIAGEN, Hilden, Germany). Subsequently, bead beating was performed using a SpeedMill PLUS (Analytik Jena, Jena, Germany) for 45 s at 60 Hz. Samples were then heated to 95 ◦C for 5 min and centrifuged for 1 min at 10.000 rpm. The resulting supernatant was transferred to a 1.5 mL microcentrifuge tube, which was placed in the QIAcube for follow-up automated DNA isolation according to the manufacturer’s instructions. Isolated DNA was used to amplify the hypervariable regions V1–V2 of the *16S rRNA* gene using the primer pair 27F (AGAGTTTGATCCTGGCTCAG)-338R (TGCTGCCTCCCGTAGGAGT) in a dual-barcoding approach according to Caporaso et al. [3]. A total of 3 µg of DNA were used for amplification, and PCR products were verified by electrophoresis in agarose gel. PCR products were normalized using the SequalPrep Normalization Plate Kit (Thermo Fisher Scientific, Waltham, MA, USA) pooled equimolarly and sequenced on the Illumina MiSeq v3 2 × 300 bp (Illumina Inc., San Diego, CA, USA).

- 1. **Feeding Mode Analysis: Supplementation of Fresh Nutrients**

Bacterial DNA isolation and *16S rRNA* gene sequencing were performed by Procomcure Biotech, Bergheim, Austria. Stool samples were thawed, and DNA was isolated using the SphaeraMag^®^ Genomic DNA Fecal Purification Kit (Procomcure Biotech, Austria). Samples have been processed individually in tubes to minimize the risk of cross-contamination. Library preparations and sequencing was done according to the Illumina protocol. Hypervariable regions V1-V2 were amplified using the primers 27F (AGAGTTTGATCCTGGCTCAG) and 375R (CTGCTGCCTYCCGTA) containing Illumina adapters. Sequencing was done with an Illumina NextSeq2000 instrument according to the application note.

- 1. **Establishment of the Sarcopenia Model and Glutamine Supplementation**

Bacterial DNA isolation and *16S rRNA* gene sequencing were performed by the Core Facility of Molecular Biology at the Center for Medical Research, Medical University Graz, Graz, Austria. Bacterial cell pellets were subjected to mechanical and enzymatic lysis using the QIAsymphony DSP Virus/Pathogen Mini Kit (QIAGEN, Hilden, Germany) with a protocol adapted for stool samples. Automated DNA isolation was performed in batches of 24 samples on a QIAsymphony device (QIAGEN, Hilden, Germany). For *16S rRNA* PCR amplification, the FastStart High Fidelity PCR system with dNTPack (Roche, Mannheim, Germany) was used to amplify the V1–V2 hypervariable region with primers 27F (AGAGTTTGATCCTGGCTCAG) and 375R (CTGCTGCCTYCCGTA) containing Illumina adapters. PCR products were normalized using NormPlate (Life Technologies, Frederick, MD, USA) and subjected to indexing PCR with Illumina index primers. Indexed PCR products were purified using the QIAquick Gel Extraction Kit (QIAGEN, Hilden, Germany). Library quality was assessed using a BioAnalyzer 2100 (Agilent, Waldbronn, Germany). The final library, including quality controls, was sequenced on an Illumina MiSeq desktop sequencer (Eindhoven, Netherlands) using the MiSeq Reagent Kit v3 (600 cycles) according to the manufacturer’s instructions.

## Statistical Analysis

Processed sequence data were further analysed using R (R Core Team, 2023, version 4.3.0) through the RStudio interface using CBmed Microbiome Analysis Platform. Alpha diversity analysis was quantified by the Richness, Shannon index, Inverse Simpson, Evenness, and Phylogenetic Diversity indices. Beta diversity was examined by principal coordinate analysis (PCoA) combined with beta diversity similarity metrics: the unique fraction metrics (*unifrac*), weighted unique (*wunifrac*) fraction matrix, Bray-Curtis’ dissimilarity (*bray*), and Jaccard (*jaccard*). The results were evaluated using Permutational Multivariate Analysis of Variance (PERMANOVA) using the *vegan* package (version 2.6.4) in R [4]. Subsequently, Linear discriminant analysis Effect Size (LEfSe) analysis was performed to identify features that exhibit differential abundance between different study groups and to determine their effect sizes, using the *microbiomeMarker* package (version 1.8.0) [5]. A Linear Discriminant Analysis (LDA) cut-off value of 3 was applied uniformly to all groups, enabling the identification of substantial differences in abundance with increased precision. Subsequently, a linear model was employed to ascertain the statistical significance of the obtained results using the *lme4* package (version 1.1.35.2) [6]. Figures were created using the *ggplotify* package (version 0.1.2) [7], heatmaps were created using *microViz* package (version 0.12.0) [8]. A level of *p* < 0.05 was used as the threshold for determining statistical significance.

**SUPPLEMENTARY REFERENCES**

[1] Reisinger, A.C. *et al.* (2021). Branched-Chain Amino Acids Can Predict Mortality in ICU Sepsis Patients. *Nutrients*. https://doi.org/10.3390/nu13093106.

[2] Pang, Z. *et al.* (2024). MetaboAnalyst 6.0: towards a unified platform for metabolomics data processing, analysis and interpretation. *Nucleic Acids Research*. https://doi.org/10.1093/nar/gkae253.

[3] Caporaso, J.G. *et al.* (2012). Ultra-high-throughput microbial community analysis on the Illumina HiSeq and MiSeq platforms. *The ISME journal*. https://doi.org/10.1038/ismej.2012.8.

[4] Oksanen, J. *et al.* (2022). vegan: Community Ecology Package.

[5] Cao, Y. *et al.* (2022). microbiomeMarker: an R/Bioconductor package for microbiome marker identification and visualization. *Bioinformatics (Oxford, England)*. https://doi.org/10.1093/bioinformatics/btac438.

[6] Bates, D. *et al.* (2015). Fitting Linear Mixed-Effects Models Using lme4. *Journal of Statistical Software*. https://doi.org/10.18637/jss.v067.i01.

[7] Yu, G. (2021). Convert Plot to “grob” or “ggplot” Object.

[8] Barnett, D.J. m *et al.* (2021). microViz: an R package for microbiome data visualization and statistics. *Journal of Open Source Software*. https://doi.org/10.21105/joss.03201.
